# Progressive sleep disturbance in various transgenic mouse models of Alzheimer’s disease

**DOI:** 10.3389/fnagi.2023.1119810

**Published:** 2023-05-19

**Authors:** Victor J. Drew, Chanung Wang, Tae Kim

**Affiliations:** ^1^Department of Biomedical Science and Engineering, Gwangju Institute of Science and Technology, Gwangju, Republic of Korea; ^2^Department of Neurology, Washington University School of Medicine, St. Louis, MO, United States

**Keywords:** sleep deterioration, Alzheimer’s disease, mouse model, amyloid beta, tau

## Abstract

Alzheimer’s disease (AD) is the leading cause of dementia. The relationship between AD and sleep dysfunction has received increased attention over the past decade. The use of genetically engineered mouse models with enhanced production of amyloid beta (Aβ) or hyperphosphorylated tau has played a critical role in the understanding of the pathophysiology of AD. However, their revelations regarding the progression of sleep impairment in AD have been highly dependent on the mouse model used and the specific techniques employed to examine sleep. Here, we discuss the sleep disturbances and general pathology of 15 mouse models of AD. Sleep disturbances covered in this review include changes to NREM and REM sleep duration, bout lengths, bout counts and power spectra. Our aim is to describe in detail the severity and chronology of sleep disturbances within individual mouse models of AD, as well as reveal broader trends of sleep deterioration that are shared among most models. This review also explores a variety of potential mechanisms relating Aβ accumulation and tau neurofibrillary tangles to the progressive deterioration of sleep observed in AD. Lastly, this review offers perspective on how study design might impact our current understanding of sleep disturbances in AD and provides strategies for future research.

## Introduction

Alzheimer’s disease (AD) is the most common cause of dementia, with an estimated global prevalence of 32 million cases (Gustavsson et al., 2023). Although individual experiences with AD differ from patient to patient, sleep disruption is increasingly recognized as a potential early indicator general symptom of AD, and one with serious implications on quality of life. Previous research indicates that sleep disturbances occur in 19–44% of patients with AD (Carpenter et al., 1996). Patients in early stages of AD report diminished sleep in the form of sleep fragmentation, nighttime awakenings, daytime drowsiness, and nocturnal wandering, also referred to as “sundowning” (Vitiello and Prinz, 1989; Vitiello et al., 1990; Holth et al., 2017b; Wang and Holtzman, 2020).

In AD, amyloid beta (A β ) accumulates and aggregates to form plaques, which contribute either directly or indirectly to other pathologies of AD, including gliosis, cognitive impairment, and the formation of tau neurofibrillary tangles (NFTs; Hardy and Higgins, 1992). Altered sleep behavior may serve as an early indicator of AD and can often precede other clinical manifestations. In fact, sleep deprivation and other sleep impairments, such as sleep fragmentation or an altered sleep/wake cycle, are believed to be related to increased levels of Aβ in the brain (Ju et al., 2014; Minakawa et al., 2017; Brzecka et al., 2018). Furthermore, several studies suggest that Aβ or tau pathologies may be involved in the deterioration of sleep quality and quantity. It has been demonstrated that as A β accumulates during the progression of AD, vigilance states shift in favor of increased wakefulness, mitigating the clearance of A β (Cedernaes et al., 2017). Sleep disruption has been posited as a potential risk factor for cognitive decline (Gagnon et al., 2009) making sleep a critical area of research in the field of neurodegenerative disorders.

This review examines the sleep behaviors exhibited by various mouse models of AD as reported in previous studies. The individual characteristics of each mouse line may offer insight into how specific pathological features of AD contribute to the disruption of sleep, and vice versa.

### Transgenic mouse models of AD

The use of mouse models provides researchers an abundance of advantages in the form of fast growth and developmental processes, minimal maintenance requirements, ease of handling, high yield of offspring, well-documented physiology and anatomy, as well as established techniques for precise genetic modification.

Most mouse models of AD are produced through the introduction of transgenes. Different AD mouse models exhibit different phenotypes, and of varying degrees, to model key aspects of the neurodegenerative disease (Hall and Roberson, 2012). AD mouse lines consist of single and multi-transgenic models (van Leuven, 2000; Myers and McGonigle, 2019). The generation of AD mouse models is often achieved via the introduction of mutations to the amyloid precursor protein (APP) gene, the presenilin 1 (PS1) gene, or the insertion of a human tau (hTau) transgene (Paulson et al., 2008). AD models have successfully replicated several pathologies associated with AD including the formation of Aβ plaques or tau NFTs, inflammation, gliosis, axonopathy, loss of neurons and synaptic damage, several forms of cognitive dysfunction, behavioral deficits and more.

Sleep is a complex phenomenon. While no single mouse model perfectly replicates sleep as it occurs in humans with AD, the models can still reproduce comparable sleep characteristics triggered by the genetically modified phenotypic qualities introduced into the mouse models. However, the order, as well as the ages at which the AD pathologies and their corresponding sleep disruptions manifest (Supplementary Table 1) vary depending on the mutations and chronology of other pathologies specific to the mouse model being studied. Tables have been included to describe progressive changes in vigilance state durations (Supplementary Table 2), bout lengths (Supplementary Table 3), bout counts or episodes (Supplementary Table 4) and power spectra or power density (Supplementary Table 5).

### APP-based mouse models of AD

#### APP23

The APP23 mouse model utilizes the Thy1 promoter to drive the 7-fold overexpression of human APP (hAPP) containing Swedish (K670N/M671L) mutation. APP23 mice display memory deficits as early as 3 months (Van Erum et al., 2019), several months before the earliest reported manifestations of Aβ pathology and tau hyperphosphorylation, which were both first observed at 6 months (Sturchler-Pierrat et al., 1997). Early Aβ pathology can be observed in the frontal cortex, subiculum and hippocampus, accompanied by small quantities of activated microglia (Stalder et al., 1999), and progresses to the thalamus and olfactory nucleus at later ages (Sturchler-Pierrat et al., 1997). By 14–18 months, prominent plaque formation occurs in the neocortex and hippocampus, coinciding with a significant loss of CA1 pyramidal neurons (Calhoun et al., 1998).

Prior to the presence of Aβ pathology, APP23 mice were found to display a significant increase in wake duration along with an increase in wake beta power compared to wild type mice (Van Erum et al., 2019). During the early stages of Aβ deposition (6 months), APP23 mice exhibit subtle indications of sleep disturbances in the form of brief awakenings during light phase as well as a significant decrease in wake and non-rapid eye movement (NREM) delta power (Van Erum et al., 2019). NREM delta power, also referred to as slow-wave activity, is a measure of homeostatic sleep pressure. Reductions in NREM delta power may be an indication of sleep inefficiencies or an inability to sustain deep sleep. By 12 months, the shift towards faster spectral frequencies advanced to include multiple frequency bands and affected all vigilance states (Van Erum et al., 2019). Increased wake durations, and decreased NREM and REM sleep durations accompanied the changes in power spectra (Van Erum et al., 2019). Interestingly, the prolonged wake durations, and reduced NREM and REM sleep durations only manifested during the dark (active) phase and not the light phase, suggesting hyperactive behavior reminiscent of the Sundowning phenomenon, in which individuals with AD demonstrate elevated activity or agitation during late afternoon or early morning (Van Erum et al., 2019).

#### App^NL-G-F^ knock-in

The App^NL-G-F^ knock-in line is a recently developed mouse model of AD containing a knocked-in human APP gene with the Swedish, Beyreuther/Iberian (I716F) and Arctic (E693G) mutations driven by the endogenous human APP promoter (Saito et al., 2014; Maezono et al., 2020). Unlike many other mouse models, the App^NL-G-F^ line is capable of generating elevated levels of Aβ without overexpressing APP, which may provide an advantage for AD sleep research by reducing unintended interactions that may alter sleep in a way that differs from AD patients with endogenous APP levels (Maezono et al., 2020). Homozygous App^NL-G-F^ mice aggressively generate Aβ plaques and gliosis starting at 2 months (Nilsson et al., 2014), subcortical amyloidosis at 4 months, and almost reach plaque saturation by 7 months (Saito et al., 2014). Memory impairment as determined by the Y-maze (Saito et al., 2014), the Morris water maze (MWM; Mehla et al., 2019), and fear conditioning tasks (Mehla et al., 2019), occurs at 6 months, coupled with reports of anxiolytic-like behavior (Sakakibara et al., 2018). Memory deficits revealed by object recognition tasks do not appear until 9 months (Mehla et al., 2019). In contrast to the Y-maze findings reported at 6 months in Saito et al. (2014), performance in the Barnes maze task revealed subtle impairments in spatial learning ability at 8 months, but an overall retention of memory functions (Sakakibara et al., 2018). Contextual fear conditioning tasks also revealed intact learning and memory at 15–18 months (Sakakibara et al., 2018). Homozygous App^NL-G-F^ mice display elevated reactivity to pain stimuli at 15–18 months (Sakakibara et al., 2018). Moreover, a battery of tests showed that 10-month-old App^NL-G-F^ mice exhibited deficits in social interactions, object recognition, and working memory, as well as displayed signs of depression (Locci et al., 2021).

At 6 months, homozygous App^NL-G-F^ mice were reported to exhibit a reduction in REM sleep duration compared to wild type mice (Maezono et al., 2020). This decrease becomes even more prominent at 12 months (Maezono et al., 2020). The change in REM sleep duration occurs before an initial decrease in NREM sleep duration, which was not observed until 12 months (Maezono et al., 2020). However, the ratio of REM to NREM sleep is reduced at both 6 and 12 months in homozygous App^NL-G-F^ mice, similar to that reported in Schneider et al. (2014), observed in 9-month-old 5XFAD mice (Schneider et al., 2014). In addition to changes in durations, homozygous App^NL-G-F^ mice also displayed decreases in wakefulness and NREM bout counts at 6 months and NREM bout counts at 12 months, as well as increases in NREM delta power and reductions in REM theta power (Maezono et al., 2020). Theta oscillations during REM are believed to be associated with memory consolidation (Boyce et al., 2016).

#### J20

The J20 mouse line overexpresses hAPP containing the Swedish and Indiana (V717F) mutations with transgene expression driven by the PDGF-β promoter. Punctate Aβ can be identified in the hippocampus of these mice as early as 1 month (Hong et al., 2016), followed by significant loss of hippocampal synapses (Hong et al., 2016), gliosis (Wright et al., 2013), elevated neuronal differentiation (López-Toledano and Shelanski, 2007) and neuronal loss (Wright et al., 2013), occurring at approximately 3 months. Initial spatial learning and memory deficits manifest emerged at 4 months, demonstrated by the MWM (Cheng et al., 2007) and radial arm maze (Wright et al., 2013). Interestingly, plaques were among the last pathological changes, reaching significantly elevated levels in the hippocampus at roughly between 7 and 8 months (López-Toledano and Shelanski, 2007; Wright et al., 2013).

The first changes in sleep behavior of J20 mice occur at 11–12 months, after extensive amyloid pathology and cognitive impairment. However, it is possible that sleep research on J20 mice has yet to be conducted at an earlier age. Sleep changes at 11–12 months are limited to a reduction in REM sleep during the second half (6-h bin) of the 12-h light phase, as well as decreased NREM delta power, increased NREM theta and sigma power and time-dependent alterations in NREM gamma power compared to wild type littermates (Filon et al., 2020).

#### PDAPP

The PDAPP mouse model is among the earliest mouse models of AD. This mouse model overexpresses hAPP containing the Indiana mutation, utilizing the PDGF-β promoter (German et al., 2005). From an early age, PDAPP mice demonstrate cognitive and memory deficits as assessed by the radial maze and barpress learning task at 3 months (Dodart et al., 1999), and MWM at 4 months (Hartman et al., 2005). At 4–5 months, PDAPP mice exhibit diminished long-term potentiation (LTP), followed by initial Aβ pathology arising at 6–9 months (Games et al., 1995). Gliosis coincides with the appearance of plaques (Games et al., 1995). Analysis of synaptophysin immunoreactivity revealed a reduction of synaptic density in the dentate gyrus by 9 months (Games et al., 1995).

Sleep dysfunction in PDAPP mice begins at approximately the same age as cognitive and memory decline, as previously reported. Young PDAPP mice, aged 3–5 months, display a partial decrease in REM sleep duration, isolated to specific segments of the light/dark phase (Huitrón-Reséndiz et al., 2002). Young PDAPP mice also displayed increased latency to the first REM sleep onset during both the dark and light periods (Huitrón-Reséndiz et al., 2002). At 20–26 months, PDAPP mice demonstrate similar REM sleep durations to wild type mice. However, PDAPP mice show time-dependent changes in sleep behavior with reduced NREM sleep along with increased wake durations during the latter half of the dark phase, and enhanced NREM sleep with reduced wake durations throughout most of the light phase compared to wild type mice (Huitrón-Reséndiz et al., 2002). Additionally, PDAPP mice display longer NREM and REM bout lengths during light phase, as well as fewer REM bout counts during dark phase (Huitrón-Reséndiz et al., 2002).

#### Tg2576

The Tg2576 mouse line utilizes a hAPP transgene with the Swedish mutation, driven by the hamster prion protein (PrP) promoter. This mouse model generates a reduction of outer dentate gyrus layer and hippocampal dendritic spine density at 4 and 4.5 months of age, respectively (Lanz et al., 2003; Jacobsen et al., 2006). Tg2576 mice demonstrate initial memory deficits at 6 months, as determined by MWM by 6 months (Jacobsen et al., 2006). Aβ plaques can be found in the subiculum, presubiculum and cortex in transgenic mice at 11–13 months (Hsiao et al., 1996). Gliosis was associated with plaque formation (Frautschy et al., 1998). Prior to any known cognitive or molecular deficits, Tg2576 mice are reported to form of interictal spike-like potentials starting at 5 weeks (Kam et al., 2016). These spikes occurred primarily during REM sleep (Kam et al., 2016). One study found that from 6 to 12 months, Tg2576 mice show reductions in REM sleep duration and REM sleep bout lengths compared to wild type mice (Zhang et al., 2005). The reductions in REM sleep were associated with damage to the cholinergic neurons of the pedunculopontine tegmentum (PPT), a region of the brainstem associated with REM sleep regulation (Zhang et al., 2005). Tg2576 mice exhibited lower trending NREM sleep delta power at 8 months compared to wild type mice, but this difference varied as age progressed (Wisor et al., 2005). A separate study reported age-based fluctuations in NREM delta power spectra in Tg2576 mice ranging from 8 to 17 months with 8-, 11-, and 15-month groups consisting of males only and a 17-month group consisting of females only (Wisor et al., 2005). Some of the deviations in EEG power may be attributable, at least in part, to sexual dimorphisms within the Tg2576 line.

Contrasting these findings, a study conducted by Kent et al. (2019) found that 12-month-old Tg2576 mice showed no changes in time spent in wakefulness, NREM or REM sleep compared to age-matched non-transgenic littermates (Kent et al., 2018). There were also no stage-specific significant changes in wake delta, NREM delta, REM delta, or REM theta power identified in the frontal EEG spectra of Tg2576 mice (Kent et al., 2018). However, parietal EEG revealed decreased delta power during wakefulness in Tg2576 mice (Kent et al., 2018). Additionally, the transgenic mice displayed overall EEG power reductions in low frequencies (delta and theta) and power increases in higher (alpha and beta) frequencies compared to wild type mice (Kent et al., 2018). The overall power spectral changes were dependent upon EEG electrode location and vigilance stage, with spectral changes during wakefulness being most pronounced (Kent et al., 2018).

#### TgCRND8

The TgCRND8 mouse model overexpress hAPP containing the Swedish and Indiana driven by the PrP promoter, which leads to the ~5x overexpression of mutant hAPP (Hock and Lamb, 2001). TgCRND8 mice are believed to possess elevated seizure susceptibility due to the high mortality rate (at pre- and post-amyloid ages; Del Vecchio et al., 2004). Transgenic TgCRND8 mice show hippocampal brain-derived neurotropic factor deficiencies compared to wild type mice as early as 6 weeks (Francis et al., 2012). By 8 weeks, TgCRND8 mice display progressive object recognition deficits (Francis et al., 2012). Initial amyloid deposits, gliosis and inflammation appear at 2–3 months in the hippocampus and cerebral cortex and progress towards the thalamus with age (Chishti et al., 2001; Dudal et al., 2004). Amyloid pathology appears to be associated with spatial learning impairments as assessed by MWM tasks (Chishti et al., 2001). At 6 months, TgCRND8 mice exhibit a loss of hippocampal neurons (Steele et al., 2014), display synaptic deficiencies (Adalbert et al., 2009), and demonstrate impaired spatial working memory as determined by the 6-arm radial water maze (RWM; Janus, 2004; Lovasic et al., 2005). At 7 months, TgCRND8 mouse brains present extensive amyloid deposits extending throughout the cortex, hippocampus, basal forebrain and thalamus (Bellucci et al., 2006). Interestingly, at 7 months, TgCRND8 mice exhibit elevated cortical and hippocampal tau hyperphosphorylation compared to wild type mice despite expressing similar levels of total tau levels (Bellucci et al., 2007).

TgCRND8 mice appear to demonstrate their first evidence of sleep impairment at 3 months in the form of increased hyperarousal and reduced NREM sleep compared to wild type mice (Colby-Milley et al., 2015). TgCRND8 mice also exhibit alterations in oscillatory activity including decreased wake delta and increased wake beta and gamma power (Colby-Milley et al., 2015). NREM delta power remained unaltered at this age (Colby-Milley et al., 2015). TgCRND8 mice show additional alterations in oscillatory activity including decreased REM theta and increased NREM and REM gamma power (add age; Colby-Milley et al., 2015). However, TgCRND8 mice no longer display decreased wake delta at this age (Colby-Milley et al., 2015). At 11 months, transgenic mice no longer show lower REM sleep durations compared to wild type mice (Colby-Milley et al., 2015). TgCRND8 NREM gamma power also returns to levels comparable to wild type mice at 11 months (Colby-Milley et al., 2015).

### APP and PSEN double transgenic mouse models of AD

#### 5XFAD

The 5XFAD mouse model is among the most established animal models used AD research (Kang et al., 2021). This double transgenic model utilizes hAPP containing the Swedish, London and Florida (I716V) mutations, and the PS1 gene with two additional mutations (M146L and L286V), driven by the Thy-1 promotor. There are three versions of this mouse model (Tg7031, Tg7092, and Tg6799) with varying levels of APP and PS1 transgene expression, Tg6799 demonstrating the most aggressive Aβ manifestation (Oakley et al., 2006). 5XFAD mice exhibit the earliest and most intense onset of amyloid pathology (Ismeurt et al., 2020), with intraneuronal Aβ developing as early as 1.5 months and the first appearance of Aβ deposits occurring at 2 months accompanied by gliosis (Oakley et al., 2006). By 3 months, brain regions including the cortex, hippocampus and midbrain and pons exhibited axonal dilatations and the severity increased with age (Jawhar et al., 2012). Cognitive deficits, including impairments of frontal cortex-associated executive function are reported to occur at approximately 4 months (Girard et al., 2013), followed by cognitive and memory deficits as determined by Y-maze at 4–5 months (Oakley et al., 2006) and advanced amyloid pathology at 6 months (Oakley et al., 2006; Ismeurt et al., 2020). Plaque load accelerates with age before reaching saturation at around 9 months (Oakley et al., 2006) 5XFAD mice also show significant loss of pyramidal neurons in correlation with memory impairment in conjunction with advanced levels of cerebral Aβ at 9 months (Oakley et al., 2006).

Two studies (Sethi et al., 2015; Duncan et al., 2019) utilizing a noninvasive piezoelectric sleep monitoring system revealed a reduction in NREM sleep duration in 5XFAD mice at ages 4–4.5 and 4–6.5 months (Sethi et al., 2015; Duncan et al., 2019). One of those studies, separating the groups by sex, found that male 5XFAD mice did not differ from wild type males in NREM sleep durations, whereas female mice showed large decreases in NREM sleep durations compared to wild type females (Duncan et al., 2019). Sethi’s study also revealed sleep fragmentation in the form of decreased light and dark phase and total 24-h period sleep bout lengths in male 5XFAD mice aged 4–6.5 months (Sethi et al., 2015). A separate study using EEG reported observing a general decrease in delta, theta alpha, beta and gamma frequency bands in 6-month-old 5XFAD mice (Schneider et al., 2014). However, the specific vigilance states of the previously mentioned EEG changes were not reported (Schneider et al., 2014). The same study also identified a decreasing proportion of REM sleep to total sleep in an 8-day analysis (Schneider et al., 2014). A recent comprehensive study conducted by Oblak et al. (2021), showed no significant differences in percentage of total time spent in wakefulness, NREM or REM sleep, in either light or dark period when comparing 10-11-month-old male 5XFAD mice with male C57BL/6 J mice (Oblak et al., 2021). They also reported no statistical differences in delta, theta, alpha, beta and gamma frequency bands during wakefulness, NREM or REM sleep (Oblak et al., 2021).

#### APP^swe^/PS1^∆E9^

The APP^swe^/PS1^∆E9^ line is a double transgenic model containing the chimeric mouse/human APP transgene with the Swedish mutation and the human PS1 gene with a deletion of exon 9. Among the earliest reported AD pathologies in this mouse model are impaired synaptic plasticity and reduced transient LTP starting at 3 months (Volianskis et al., 2010), the initial detection of Aβ40 and Aβ42 between 3 and 4 months (Garcia-Alloza et al., 2006; Volianskis et al., 2010), and synaptic loss at 4 months (Hong et al., 2016). Aβ40 and Aβ42 increase linearly with age with no change in ratio (Volianskis et al., 2010). A noteworthy proportion (up to 65%) of transgenic APP^swe^/PS1^∆E9^ mice have also been reported to exhibit single or multiple unprovoked seizures between 3 and 4.5 months of age, whereas non-transgenic mice did not display this trait (Minkeviciene et al., 2009). APP^swe^/PS1^∆E9^ mice develop Aβ plaques in the cortex and hippocampus as early as 6 months, reaching substantial levels by 9 months (Lesuisse et al., 2001; Jankowsky et al., 2004). Plaques were associated with the presence of GFAP-positive reactive astrocytes (Kamphuis et al., 2012). In addition to plaque formations and reactive astrogliosis, APP^swe^/PS1^∆E9^ mice also displayed contextual memory impairments as demonstrated by reduced freezing behavior during a contextual fear conditioning paradigm compared to wild type controls (Kilgore et al., 2010). A separate study also reported the presence of memory deficits in transgenic APP^swe^/PS1^∆E9^ mice by 6 months, as evidenced by making significantly more errors in the RWM test (Xiong et al., 2011). It was demonstrated that neurons and dendritic segments close to plaques presented diminished activity in APP^swe^/PS1^∆E9^ mice between 9 and 11 months of age (Meyer-Luehmann et al., 2009).

The literature covering the sleep behavior observed in the APP^swe^/PS1^∆E9^ mouse model show widely varying results. According to Zhang et al. (2019), the first sleep impairments in the APP^swe^/PS1^∆E9^ model emerge at 3 months in the form decreased power spectra during NREM and REM sleep compared to wild type mice (Zhang et al., 2019). Although the range of frequencies fluctuate, APP^swe^/PS1^∆E9^ mice largely maintain these reductions through 9 months of age (Zhang et al., 2019). At 3 months, transgenic mice also displayed decreased NREM sleep durations. At 6 and 9 months, the differences in NREM sleep durations between transgenic and wild type mice varied by light/dark phase, but ultimately did not differ significantly the over 24-h recording periods. A study by Roh et al. (2012) reported diminished REM sleep durations in APP^swe^/PS1^∆E9^ mice by 9 months (Roh et al., 2012). A study by Kent et al. (2018), found frontal NREM and parietal wake power spectra shifting to higher frequencies at 8–10 months in APP^swe^/PS1^∆E9^ mice compared to wild type mice (Kent et al., 2018). Alterations to specific frequency bands included increased frontal NREM beta and gamma power, and parietal wake and NREM beta power, as well as decreased parietal wake theta and NREM delta power (Kent et al., 2018). A separate study by Kent et al. (2019), found that 12-month-old transgenic mice displayed increased beta power compared to wild type mice during wakefulness, whereas alpha and beta power increased significantly while delta power decreased in transgenic mice compared to non-transgenic mice during NREM sleep (Kent et al., 2019).

#### APP^swe^/PS1^A246E^

The APP/PS1^A246E^ mouse model overexpresses hAPP with the London (V717I) mutation and human PS1 with an A246E mutation, with both transgenes driven by the Thy1 promoter. One study reports that these mice demonstrate indications of poorer recognition memory at early as 3 months (Filali and Lalonde, 2015) and generate their earliest plaques between 6 and 9 months (Dewachter et al., 2000; Liu et al., 2002). However, it should be mentioned that another study did not observe cognitive deficits in the APP/PS1^A246E^ model until 11–12 months, as measured by MWM (Puoliväli et al., 2002). Plaque burdens accelerate quicker in female APP/PS1^A246E^ mice than males (Wang et al., 2003). By 10 months, transgenic APP/PS1^A246E^ mice exhibit a roughly 50% loss of pyramidal neurons in the CA1/2 hippocampal layer, correlating with the strong accumulation of intracellular Aβ (Casas et al., 2004).

Sleep deterioration in APP/PS1^A246E^ mouse model starts at 5 months with a significant reduction in NREM sleep duration, exclusive to the dark phase (Jyoti et al., 2010). NREM sleep deterioration is further enhanced by 12 months, affecting both light and dark phases (Jyoti et al., 2010). These mice do not appear to demonstrate a change in REM sleep durations from 5 to 12 months (Jyoti et al., 2010).

### APP, PSEN, and tau triple transgenic mouse models of AD

#### 3xTgAD

The 3xTgAD mouse is a triple transgenic model that incorporates the hAPP transgene with the Swedish mutation, human PS1 transgene with an M146V mutation, and human tau transgene with the P301L mutation, making it one of a limited number of AD models that express both, Aβ plaques and tau NFTs (Sterniczuk et al., 2010). Evidence of intracellular Aβ42 along with GFAP-positive astrocytes and tau immunoreactivity can be detected in regions of the hippocampus as early as 2 months (Mastrangelo and Bowers, 2008). 3xTgAD mice exhibit retention deficits and long-term memory impairments at 4 months (Billings et al., 2005). Plaques appear at approximately 6 months (Oddo et al., 2003b; and can eventually be observed in the extracellular posterior cortex at 15 months; Oddo et al., 2003a). Basal synaptic transmission is disrupted and long term potentiation also becomes impaired in 3xTgAD mice at 6 months (Oddo et al., 2003b). Tau pathology reaches extensive levels by 12 months (Oddo et al., 2003b).

At 6 months of age, 3xTgAD mice demonstrate increased slow wave sleep (SWS) bout lengths despite maintaining comparable SWS durations compared to wild type mice (Cushing et al., 2020). At this same age, transgenic mice display decreased sleep spindle density (Cushing et al., 2020). Sleep spindles are rhythmic bursts of 12–15 Hz (Brancaccio et al., 2020) oscillations that last between 0.5 and 3 s and are prevalent during NREM sleep (Kim et al., 2012). Sleep spindles are also thought to provide sleep stability (Kim et al., 2012), but may also have a role in memory consolidation. Unlike most AD mouse models, 3xTgAD mice do not show changes in wakefulness, NREM or REM sleep durations at 18 months of age (Kent et al., 2018). Furthermore, 3xTgAD mice do not exhibit changes in frontal EEG power spectra at 18 month compared to wild type mice (Kent et al., 2018).

#### PLB1

The PLB1 mouse line is a triple transgenic model of AD containing hAPP with the London and Swedish mutations, human PS1 with an A246E mutation and human microtubule-associated protein tau (MAPT) P301L and R406W mutations (Bouter and Bouter, 2019). PLB1 mice display their first indications of memory decline at 4 months demonstrating decreased ability to discriminate between objects as determined by the object recognition tasks (Ryan et al., 2013). At 5 months, PLB1 mice exhibit impaired social recognition memory. A reduction in long-term plasticity paralleled by hyperphosphorylated tau can be identified in the hippocampus at 6 months, however, this model is not believed to express overt NFT pathology (Platt et al., 2011). One study identified a discrete number of hippocampal and cortical extracellular plaques present in PLB1 mice at 6 months (Koss et al., 2013).

Studies examining EEG spectra output of PLB1 mice have slightly varying results. A study conducted by Platt et al. (2011) found increases in wake and NREM delta power in PLB1 mice compared to wild type mice at 5 months, whereas a separate study (Jyoti et al., 2015) reported no difference in these areas. Both studies, however, identified an increase in REM theta at 5 months (Platt et al., 2011; Jyoti et al., 2015). At later ages, both studies also recognized varying degrees of increased delta power during wakefulness NREM sleep, and increased theta power during NREM and REM sleep (Platt et al., 2011; Jyoti et al., 2015). Reports on sleep/wake durations also yielded conflicting findings. Between the ages of 5 to 21 months, Jyoti et al. (2015) did not observe any change in NREM sleep duration for PLB1 mice compared to wild type mice, but did detect decreases in REM sleep duration restricted to the light phase at varying ages (Jyoti et al., 2015). Conversely, Platt et al. (2011) reported a consistently lower NREM sleep duration in 5-month-old and 12-month-old PLB1 mice compared to age-matched wild type mice, with no differences in REM sleep duration (Platt et al., 2011).

### Other mouse models of AD

#### APPSwDI/NOS2 −/− (CVN-AD)

The CVN-AD mouse model is a bigenic line that incorporates the hAPP transgene with the Swedish, Iowa (D694) and E693Q mutations, under the control of the Thy1 promoter, with mouse nitric oxide synthase (NOS2) knocked-out. These mice demonstrate an early onset of amyloid plaques at approximately 3 months, and behavioral changes, loss of hippocampal cells, and phosphorylated tau between 5.5 and 8 months (Turner, 2021). CVN-AD mice also exhibit significantly elevated reactive astrocytes and cortical microglia at 8–9 months compared to age-matched controls (Nwafor et al., 2021). By 12 months, CVN-AD mice display deficits in spatial learning and memory as determined by radial-arm water maze performance. At this age, extensive tau hyperphosphorylation is associated with dense Aβ deposition in transgenic, but not wild type mice (Wilcock et al., 2008).

With regards to sleep impairments, CVN-AD mice show relatively similar behavior to age-matched wild type mice (Nwafor et al., 2021). At 8–9 months, transgenic CVN-AD mice display no changes in sleep duration during light phase with an insignificant (*p* = 0.087) reduction in sleep duration during the dark phase (Nwafor et al., 2021). CVN-AD mice also show no differences in sleep bout duration during light or dark phases compared to wild type mice (Nwafor et al., 2021). Interestingly, CVN-AD mice demonstrated a significant increase in bout counts, but this difference was limited to the dark phase (Nwafor et al., 2021).

#### P301S tau (PS19)

The P301 Tau mouse line (also known as PS19) was designed to model the effects of tauopathy through the progression of AD. This mouse model utilizes a human MAPT transgene containing the P301S mutation. P301S Tau mice develop microgliosis in the hippocampus as early as 3 months followed by NFTs in the neocortex, amygdala, hippocampus, and other regions of the brain by 5 months (Yoshiyama et al., 2007). Synaptic deterioration occurs by at 6 months (Yoshiyama et al., 2007) along with the first evidence of memory impairment, as determined by the MWM task (Takeuchi et al., 2011). Loss of hippocampal neurons occurs at 8 months (Yoshiyama et al., 2007).

The first indications of sleep impairment in the P301S Tau mouse model occur at 6 months. At this age, transgenic mice demonstrate an apparent increase in NREM delta power compared to wild type mice (Holth et al., 2017a). By 9 months, transgenic mice also display higher NREM and REM theta, potentially indicating a spectral shift toward slower frequencies (Holth et al., 2017a). Interestingly, these trends reverse at 11 months in which wild type mice exhibit higher levels of wake and NREM delta power and REM theta power (Holth et al., 2017a). Changes in sleep architecture of P301S Tau mice began at 9 months with a decrease in REM sleep duration. The diminished REM sleep duration persisted into 11 months and was accompanied by a reduction in NREM duration (Holth et al., 2017a). Transgenic P301S Tau mice also display a longer NREM bout lengths specific to 9 months, and shorter REM bout lengths, specific to 11 months, compared to age-matched controls (Holth et al., 2017a). Remarkably, P301S Tau mice exhibit reduced NREM and REM bout lengths at 9 months, which is sustained at 11 months (Holth et al., 2017a).

#### rTg4510

The rTg4510 mouse model expresses human tau containing the P301L mutation. This mouse model does not generate Aβ pathology. rTg4510 mice begin to demonstrate gliosis (Helboe et al., 2017) and spatial memory deficits beginning as early as 2.5 months, determined by the MWM task (Ramsden et al., 2005). These cognitive impairments increase in severity by 4 months (Ramsden et al., 2005). rTg4510 mice also initiate tau pathology at 2.5 months, which develop into mature NFTs readily detectable in limbic structures by 5 months (Ramsden et al., 2005). LTP declines by 4.5 months in the CA1 region of the hippocampus (Hoover et al., 2010). At 5.5–8.5 months, rTg4510 mice show loss of neurons in the CA1 region of the hippocampus (Ramsden et al., 2005). Helboe et al. (2017) reports observing synaptic loss and neurodegeneration in the CA1 region of the hippocampus of rTg4510 mice at approximately 7.5 months (Helboe et al., 2017).

Holton et al. (2020) conducted sleep study of the rTg4510 mouse model from 20 to 44 weeks of age at 4-week intervals, evaluating vigilance state durations, bout lengths, bout counts and power spectral analysis. They found that rTg4510 mice demonstrate a progressive deterioration of EEG power spectra with a steady decrease in NREM delta and theta power from roughly 4.5 months to 10 months, as well as a reduction in total EEG power over the same period (Holton et al., 2020). Throughout most ages recorded between 5.5–10 months, rTg4510 mice exhibit shorter REM bout lengths compared to wild type mice, while total sleep bout lengths remain unchanged (Holton et al., 2020). During the same time frame, rTg4510 mice showed fewer REM sleep bout counts compared to wild type mice, primarily during dark phase, while total sleep bout counts remain unchanged (Holton et al., 2020). Remarkably, it is not until 6.5 months that rTg4510 mice displayed a reduction in NREM sleep duration, which also occurred primarily during dark phase and at every age examined except 9 months (Holton et al., 2020).

#### SAMP8

Unlike the APP, PSEN and tau transgenic models, the Senescence Accelerated Mouse-Prone 8 (SAMP8) mouse model is a spontaneously occurring mouse line that exhibits characteristics of accelerated aging with relevance to the altered gene expression and protein abnormalities observed in AD (Butterfield and Poon, 2005). Reactive microglia can be observed as early as 1–2 months and became more prominent with advancing age in SAMP8 mice (Kawamata et al., 1997). By 2 months, the neuronal count of SAMP8 mice is decreased compared to young SAMP1 mice (Kawamata et al., 1997). SAMP8 mice show of age-related impairment in learning and memory at 2 months, as demonstrated by increased latency during a MWM task (Miyamoto et al., 1986). From 6 months, SAMP8 mice exhibit Aβ plaques in the hippocampus that increase with age (del Valle et al., 2010). At 12 months, SAMP8 mice express major changes in the LTP pathway involving mitogen-activated protein kinase and calcium signaling compared to control mice, which might contribute to cognitive impairments observed in aged mice (Armbrecht et al., 2014).

With regards to sleep disturbance, SAMP8 mice demonstrate decreases in NREM and REM sleep durations at 4 months. These reductions are isolated to the light phase (Beuckmann et al., 2021).

### Sleep deterioration trends in mouse models of AD

Sleep deterioration has been reported in most established mouse models of AD (Table 1 and Supplementary Tables 1–5). The forms of impairment vary by model and may be dependent upon factors such as age, sex, and the specific AD hallmark(s) expressed by each model. The sleep impairment occurring the greatest frequency in AD mouse lines appears to be the reduction of NREM sleep duration and concurrent increase in wakefulness, which has been reported in homozygous App^NL-G-F^, TgCRND8, AβPP^swe^/PS1^∆E9^, PLB1, and APP23 mice among others (Supplementary Table 2; Jyoti et al., 2010; Platt et al., 2011; Roh et al., 2012; Colby-Milley et al., 2015; Holth et al., 2017a; Van Erum et al., 2019; Zhang et al., 2019; Holton et al., 2020; Maezono et al., 2020; Beuckmann et al., 2021). A decrease in REM sleep duration appearing in at least one age group was another highly consistent form of sleep deterioration present in most AD models observed, appearing in 10 of the 15 mouse models characterized in this review. To our knowledge, no mouse model of AD has been reported to demonstrate an increase in REM duration at any age. As with sleep research in human AD subjects, results among the various mouse studies occasionally conflict with one another. Certain AD models, such as the homozygous App^NL-G-F^, PDAPP, and P301S tau models are reported to express decreased REM sleep durations prior to exhibiting altered wakefulness or NREM sleep durations (Huitrón-Reséndiz et al., 2002; Holth et al., 2017a; Maezono et al., 2020). These observations are consistent with human studies that report impaired REM sleep in preclinical or early stage AD patients suggesting that REM sleep impairment may serve as an early biomarker for AD (BONANNI et al., 2005). However, these findings are contradicted by mouse studies demonstrating diminished REM sleep occurring only after NREM sleep is significantly reduced, if at all (Jyoti et al., 2010; Van Erum et al., 2019; Zhang et al., 2019; Holton et al., 2020). Similarly, there are human studies that identify decreased SWS duration in individuals with mild-to-moderate AD, prior to any significant decrease in REM duration indicating variation among human studies (Vitiello et al., 1990). Interestingly, changes in sleep duration manifest with greater prominence during the dark (active) period in mouse models of AD (Jyoti et al., 2010; Colby-Milley et al., 2015; Sethi et al., 2015; Van Erum et al., 2019; Holton et al., 2020). This may be the result of a bidirectional interaction between sleep disturbance and elevated A β levels (Xie et al., 2013; Ju et al., 2014). In other words, at night, sleep disturbances increases A β due to the less efficient glymphatic system during wakefulness (Xie et al., 2013) and enhanced neuronal activities (Yuan and Grutzendler, 2016), whereas the increased A β , in turn, disturbs sleep (Özcan et al., 2020). This vicious cycle may lead to severe disruption of sleep in Alzheimer’s disease at night. In addition, the hyperactive behavior reported in AD mice may be comparable to the increased activity during the active periods of late afternoon or early morning characteristic of human AD patients with Sundowning syndrome (Colby-Milley et al., 2015; Van Erum et al., 2019).

**Table 1:** Summarization of sleep disturbances in various mouse models of Alzheimer’s disease compared to age-matched controls.

Mouse model	Mutations	Sleep disturbance	References
APP-based models			
APP23	· Swedish (APP)	↓ NREM and REM duration	Van Erum et al. (2019)
		↓ NREM delta power	
App^NL-G-F/NL-G-F^	· Swedish, Iberian, Arctic (APP)	↓ NREM and REM duration	Maezono et al. (2020)
		↓ REM length	
		↓ NREM count	
		↑ NREM delta power	
J20	· Swedish, Indiana (APP)	↓ REM duration	Filon et al. (2020)
		↓ NREM delta power	
PDAPP	· Indiana (APP)	↓ REM duration	Huitrón-Reséndiz et al. (2002)
		↑ NREM and REM length	
		↓ REM count	
Tg2576	· Swedish (APP)	↓ REM duration	Wisor et al. (2005), Zhang et al. (2005), Kent et al. (2018)
		↓ REM length	
TgCRND8	Swedish, Indiana (APP)	↓ NREM and REM duration	Colby-Milley et al. (2015)
APP/PSEN-based models			
AβPP^swe^/PS1^A246E^	· Swedish (APP)· A246E (PSEN1)	↓ NREM duration	Jyoti et al. (2010)
AβPP^swe^/PS1^∆E9^	· Swedish (APP)· ∆E9 (PSEN1)	↓ NREM and REM duration↓ NREM delta power	Roh et al. (2012), Kent et al. (2018), Kent et al. (2019), Zhang et al. (2019)
5XFAD	· Swedish, London, Florida (APP) · M146L, L286V (PSEN1)	↓ Total sleep duration ↓ Total sleep length	Roh et al. (2012), Kent et al. (2018), Kent et al. (2019), Zhang et al. (2019)
APP/PSEN/TAU-based models			
3xTgAD	· Swedish (APP) · M146V (PSEN1) · P301L (MAPT-TAU)	No changes	Kent et al. (2018)
PLB1	· Swedish, London (APP)	↓ NREM and REM duration	Platt et al. (2011), Jyoti et al. (2015)
	· A246E (PSEN1)	↑ NREM delta power	
	· P301L, R406W (MAPT-TAU)		
Others			
APPSwDI/NOS2 −/− (CVN-AD)	· Swedish, Iowa, Dutch (APP)	↑ Total sleep count	Nwafor et al. (2021)
	· NOS2 KO		
P301S Tau (PS19)	· P301S (MAPT-TAU)	↓ NREM and REM duration	Holth et al. (2017a,b)
		↑ NREM length	
		↓ REM length	
		↓ NREM and REM count	
rTg4510	· P301L (MAPT-TAU)	↓ NREM duration	Holton et al. (2020)
		↓ REM length	
		↓ REM count	
		↓ NREM delta power	
SAMP8	· Spontaneous	↓ NREM and REM duration	Beuckmann et al. (2021)

Bout lengths and bout counts are useful metrics to indicate the presence of sleep fragmentation. Of the mouse studies that provide data on vigilance state durations (Supplementary Table 2) and bout lengths (Supplementary Table 3), changes in durations of a particular vigilance state occasionally preceded changes in bout lengths of that state (Van Erum et al., 2019; Maezono et al., 2020). However, more frequently, alterations in durations and bout lengths of at least one vigilance state (not necessarily the same one) occurred simultaneously within the mouse model observed (Zhang et al., 2005; Colby-Milley et al., 2015; Sethi et al., 2015; Holth et al., 2017a; Duncan et al., 2019; Holton et al., 2020). The most frequent changes to bout lengths included increased wake bout lengths (Colby-Milley et al., 2015; Holth et al., 2017a; Maezono et al., 2020) and decreases in REM bout lengths (Zhang et al., 2005; Holth et al., 2017a,b; Maezono et al., 2020). The changes in bout lengths of the different vigilance states appeared to vary more so by age than by sex, with REM sleep bout lengths occasionally shortening in older mice (Zhang et al., 2005; Holth et al., 2017a; Maezono et al., 2020) and more severe deterioration occurring in female mice than in males (Sethi et al., 2015). Unlike the change vigilance state duration changes observed in many AD mouse models, changes in bout lengths between wild type and transgenic mice did not appear to be influenced by light or dark phase.

In humans, interruptions to the sleep cycle would result in increased wake and sleep episodes. Counterintuitively, sleep patterns of AD mice demonstrate decreases in wake, NREM and/or REM episodes compared to age-matched controls (Supplementary Table 4; Huitrón-Reséndiz et al., 2002; Holth et al., 2017a; Holton et al., 2020; Maezono et al., 2020). The specific vigilance states showing change vary by mouse model. In human AD, the decrease in REM duration in particular is accounted for by the reduction in REM episode length, concurrent with the maintaining of other characteristics such as number of REM episodes and REM density (Petit et al., 2004). In mice, decreases in bout counts may reflect a consolidation of sleep episodes or reduction in the ability for mice to initiate sleep. Additionally, fewer transitions between NREM and REM sleep might contribute to the reduced bout counts observed in AD mice. Interestingly, CVN-AD mice exhibited higher total sleep episode counts compared to wild type mice (Nwafor et al., 2021), which may be indicative sleep fragmentation by signifying more transitions between sleep and wakefulness. PDAPP, CVN-AD, and rTg4510 mice appear to display greater changes in bout counts during the dark period than during the light period (Huitrón-Reséndiz et al., 2002; Holton et al., 2020; Nwafor et al., 2021). In PDAPP, P301S Tau, and rTg4510 mice, sleep fragmentation in the form of increased bout counts increases with age (Huitrón-Reséndiz et al., 2002; Holth et al., 2017a; Holton et al., 2020).

While durations, bout length and bout counts serve as useful indicators of sleep quantity, EEG power spectra enable the examination of qualitative aspects of sleep. Power spectra varied heavily based on age and mouse model (Supplementary Table 5). Studies examining App^NL-G-F/NL-G-F^ and PLB1 mouse models identified an increase in wake delta power compared to controls (Platt et al., 2011; Jyoti et al., 2015; Maezono et al., 2020). This is in line with human AD studies (Petit et al., 2004). Conversely, TgCRND8 and Tg2576 mouse models demonstrated decreased wake delta power (Colby-Milley et al., 2015; Kent et al., 2018). Wisor et al. showed that the power spectra of the late stage Tg2576 model may be highly influenced by the time of day, such that transgenic Tg2576 mice displayed lower wake delta power compared to control mice during zeitgeber (ZT)0–3, no difference to controls from ZT3-6, and greater wake delta power compared to controls from ZT6 to ZT12 (Wisor et al., 2005).

NREM delta, a marker for homeostatic sleep drive in humans (Morairty et al., 2013), also differed substantially among mouse models. APP23, AβPP^swe^/PS1 Δ ^E9^ and rTg4510 exhibited a reduction in NREM delta power (Kent et al., 2018, 2019; Van Erum et al., 2019), which is consistent with the findings from human AD studies (Loewenstein et al., 1982). Conversely, App^NL-G-F/NL-G-F^, Tg2576 and PLB1 mice displayed increased NREM delta power (Wisor et al., 2005; Platt et al., 2011; Jyoti et al., 2015; Maezono et al., 2020). While wake theta has been linked to information encoding and retrieval, the function of NREM theta is less clear. However, NREM theta has been implicated in memory consolidation, in which increased NREM theta in AD patients was reported to predict higher subsequent memory performance after verbal cue testing (Schreiner and Rasch, 2015). The majority of AD mouse models show no significant difference in NREM theta power in transgenic mice compared to wild type mice. The exceptions include App^NL-G-F/NL-G-F^ mice, in which NREM theta decreases progressively (Maezono et al., 2020), and PLB1 mice, in which NREM theta increases progressively (Platt et al., 2011; Jyoti et al., 2015).

REM slowing, identified by increased REM delta and theta power and/or decreased alpha and beta power, may be associated with diminished cognition and memory in human individuals with AD (D'Atri et al., 2021). An increase in REM delta power was observed in App^NL-G-F/NL-G-F^ mice (Maezono et al., 2020) compared to control mice, whereas separate studies investigating power spectra for the PLB1 mouse model had conflicting findings (Platt et al., 2011; Jyoti et al., 2015).

Reduced spindle density (Liu et al., 2020; Weng et al., 2020), diminished K-complexes (Liu et al., 2020) and the presence of interictal spikes are additional EEG features signaling sleep impairment. Sleep spindles and K-complexes are believed to maintain sleep stability and prevent arousal from sleep, but may also play a role in memory consolidation. Interestingly, a 50-min sleep recording session of 3xTgAD mice at 6 months demonstrated a reduction of sleep spindle density concurrent with an increase in bout length without altering the proportion of total time spent in SWS (Cushing et al., 2020). This may suggest that the lengthening of the SWS bout might serve as a compensatory reaction to recover the loss of sleep spindles.

Individuals with AD are at increased risk of epileptic seizures (Jin et al., 2021). Interictal spikes are abnormal synchronous discharges from a cluster of neurons and thought to occur near or between seizure episodes. Interictal spikes can be observed during sleep as early as 5 weeks in transgenic Tg2576 mice (Kam et al., 2016), pre-plaque and before cognitive impairment. Interestingly, high frequency oscillations (HFOs; 250 ~ 500 Hz), primarily occurring during NREM sleep, were found in the hippocampus and cortex of Tg2576 mice, but are not specific to epilepsy (Lisgaras and Scharfman, 2023). However, HFOs between 80 and 200 Hz during REM sleep were reported to occur less frequently, yet be more specific to epileptogenicity (Sakuraba et al., 2016). Seizures can appear in any stage of AD, they occur at greater frequency at later stages, and often go unnoticed. The exact relationship between seizures and AD remain unclear (Raudino, 2020).

### Pathophysiological aspects associated with sleep impairment

#### Aβ and sleep impairment

AD mouse models are designed to replicate specific characteristics of AD pathology, with some models focusing on the overexpression of singular, well-established biomarkers and others models focusing on the combinations of disease factors. Divergences among models likely contribute to mechanistic differences in the manifestation of certain AD features, including sleep disruption. Many AD mouse models, such as APP23, App^NL-G-F/NL-G-F^, J20, TgCRND8 and others, demonstrate sleep/wake cycle dysfunction either after or concurrent to the earliest reports of formation of amyloid plaques, suggesting that sleep deterioration may be the result of insult or injury stemming from AD molecular biomarkers. However, Aβ distribution does not progress uniformly throughout all regions of the brain. Generally, in human AD, Aβ initially develops in regions such as the medial orbitofrontal cortex, precuneus, posterior cingulate, and isthmus cingulate cortices (Grothe et al., 2017) before progressing into subcortical regions including the thalamus, ultimately reaching the brainstem and cerebellum (Thal et al., 2002).

Sleep disruption by amyloidosis may require specific interactions between Aβ and sleep-regulating regions of the brain, such as the ventrolateral preoptic area (VLPO), medial septum, laterodorsal tegmentum (LDT), pedunculopontine tegmentum (PPT) or others (Drew et al., 2018). The REM sleep changes during the early stages of AD may be the result of damage to the cholinergic systems in the basal forebrain, which regulate REM sleep (Petit et al., 1993). Cholinergic neurons in the brainstem nuclei, such as LDT and PPT, are also involved in REM sleep and their pathological changes may also enhance REM sleep deterioration (Parvizi et al., 2001). The reductions in REM sleep observed in Tg2576 mice were believed to be caused by damage to the cholinergic neurons of the PPT, a region of the brainstem associated with REM sleep regulation (Zhang et al., 2005).

#### Tau and sleep impairment

While soluble and insoluble Aβ are often suspected of being primary contributing factors towards progressive sleep deterioration in AD, it is important to consider mouse lines that exhibit sleep impairment despite the lack of Aβ pathology, such as the P301S Tau (PS19) and rTg4510 mouse models (Holth et al., 2017a; Holton et al., 2020). Both models demonstrate initial tau pathology approximately 2 months prior to expressing altered EEG power spectra, their first indications of sleep dysfunction (Holth et al., 2017a; Holton et al., 2020). Both models also demonstrate progressive sleep deterioration in line with increasing tauopathy (Holth et al., 2017a; Holton et al., 2020). In their study, Holton et al. (2020) demonstrated that repression of tauopathy with doxycycline prevented or lessened most longitudinal EEG changes in rTg4510 mice, indicating that tauopathy may be the chief cause of the altered EEG and sleep behavior in a tau-based model of AD (Holton et al., 2020). Like Aβ, tau levels in the interstitial fluid rise with elevated neuronal activity, which suggests that increased wakefulness may elevate tau secretion, consequently enhancing tau pathology (Holth et al., 2017a). Tauopathies in the brainstem may contribute to decreased proportions of REM sleep. Conversely, a similar argument could be made for Aβ pathology in the absence of tauopathies, as many AD models, such as App^NL-G-F/NL-G-F^, J20, AβPP^swe^/PS1^∆E9^, Tg2576 and others, generate various forms of progressive sleep deterioration while lacking aberrant or modified tau expression.

#### Altered sleep behavior preceding Aβ and tau pathology

Intriguingly, several AD mouse models such as APPswe/PS1^∆E9^, PLB1, and SAMP8 demonstrate altered sleep behavior prior to the appearance of plaques (Zhang et al., 2019) or tau pathology, indicating a potential for separate mechanisms disrupting different components of sleep. One possible explanation could be the early-stage synaptic interference of sleep-regulating neurons caused by the accumulation soluble Aβ prior to the formation of insoluble plaques. A growing perspective that supports the observations in APPswe/PS1^∆E9^, PLB1, and SAMP8 mice suggests that sleep impairment may have a causal relationship with amyloid and tau levels. The chronic disruption of sleep–wake cycles leads to increased time in wakefulness. Prolonged wake durations produce increased neuronal activity, higher reactive oxygen species production, oxidative stress, increased Aβ and tau production, reduced Aβ and tau clearance, as well as neuronal death. All of these factor together potentially contribute to the destruction of sleep–wake-regulating neurocircuitry, ultimately creating a positive feedback loop (Cedernaes et al., 2017). Given the diversity of genetics within each mouse model, it is likely that more than one mechanism contributes to the progressive sleep deterioration observed in AD.

#### Abnormal electrical activity during sleep and its potential role in sleep dysfunction

As previously discussed, interictal spikes and HFO have been observed in mouse models of AD (Kam et al., 2016; Lisgaras and Scharfman, 2023). In humans, these phenomena are reported to occur more frequent during NREM sleep (Alkawadri et al., 2014; Boly et al., 2017; Lambert et al., 2018, 2020; Amiri et al., 2019), suggesting that they may affect important processes that occur during NREM sleep, such as slow wave activity and memory consolidation. Slow wave activity occurs when cortical neurons exhibit instability, oscillating between UP and DOWN states. Synchronous firing of the cortical neurons occurs during UP state, while silencing occurs during the DOWN state, both of which take place during NREM sleep (Tononi and Cirelli, 2014). Alterations in ion channels may contribute to the hyperexcitable neuronal activities in AD mouse models (Verret et al., 2012; Kim et al., 2021). Consequently, the hyperexcitability evidenced by HFOs during NREM sleep could disrupt the normal alternating pattern of the UP and DOWN states (Bragin et al., 2010, 2012; Vyazovskiy et al., 2011; Frauscher et al., 2015).

#### Neuronal groups associated with sleep disturbances in Alzheimer’s disease mouse models

**Figure 1:**
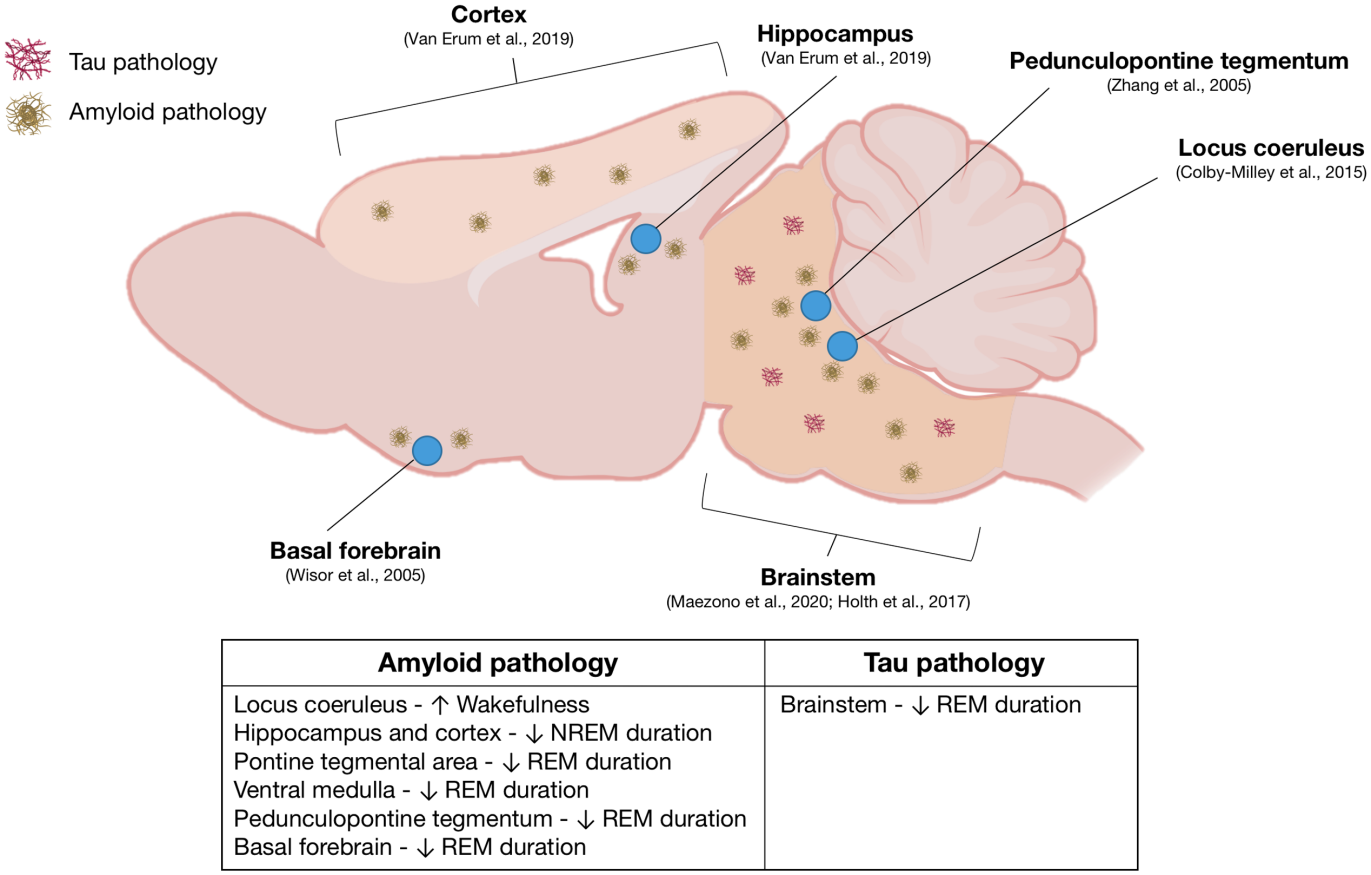
Overview of sleep-regulating regions implicated in Alzheimer’s disease sleep pathology: This figure presents a mouse brain schematic, derived from an extensive review of literature on sleep research in mouse models of Alzheimer’s disease (AD). The diagram highlights the relevant brain regions and their associated AD pathologies, specifically amyloid-beta and tau pathologies, accompanied by corresponding references. The table provides a comprehensive summary of sleep impairments correlated with each identified brain region.

Although the neurocircuitry associated with sleep disturbances in AD remains elusive, sleep disruptions in various mouse models of AD have been linked to distinct amyloid and tau pathologies occurring in sleep-regulating regions of the brain (Figure 1). In APP23 mice, sleep fragmentation coincides with the onset of plaque deposition, and both NREM and REM sleeps are significantly reduced as amyloid plaques accumulate in the neocortex and hippocampus (Van Erum et al., 2019). In APP^NL-G-F/NL-G-F^ mice, REM sleep reduction corresponds with A β accumulation in the pontine tegmental area and ventral medulla, areas essential for REM sleep regulation (Maezono et al., 2020). Interestingly, in Tg2576 mice, REM sleep impairments and cholinergic dysfunction in the PPT precede A β neurodegeneration (Zhang et al., 2005). However, another study using Tg2576 mice suggests that diminished REM sleep may result from a loss of cholinergic neurons in the basal forebrain, a region of the brain responsible for the EEG desynchronization necessary for REM sleep to occur (Wisor et al., 2005). The increased wakefulness and heightened low gamma power exhibited by TgCRND8 mice may be linked to compensatory increases in noradrenergic tone from the locus coeruleus (Colby-Milley et al., 2015). Sleep disruptions in PLB1 mice, characterized by reduced NREM sleep and fragmentation, might result from intraneuronal tau protein (Platt et al., 2011). Brainstem pathology in P301S mice, but not in PLB2-Tau models, has been suggested to cause REM sleep disruption (Holth et al., 2017a). Despite the identification of Alzheimer’s disease hallmarks in sleep-promoting brain regions, further research is required to determine the exact relationship between amyloid and tau pathologies, as well as the molecular mechanisms and neurological pathways contributing to the progression of sleep disturbances in AD mice (Kent et al., 2018).

### Methodological variation in sleep research with AD mouse models

The relationship between sleep and AD has attracted a range of experimental approaches and techniques and the findings of each study are largely dependent on the scientific strategies utilized. While the various research strategies have increased our overall knowledge about the impact of AD on sleep impairment and vice versa, the inconsistencies in the details, definitions and methodologies among the different studies may contribute to some of the discrepancies of the findings. Those variables include:

#### Different definitions of epochs and bouts

Shorter epochs result in decreased bout lengths and increased bout counts, whereas longer epochs result in increased bout lengths and decreased bout counts. This is because subtle mouse movements and/or brief shifts in EEG frequencies are more likely to be recognized as changes in vigilance states in studies using shorter epoch lengths. Furthermore, increased consecutive epoch requirements to define a bout results in fewer total bout counts and decreased consecutive epoch requirements to define a bout results in greater total bout counts.

#### Different recording periods

Mouse behavior is highly dependent on time of day. Mice are nocturnal and generally sleep more during the light period than during the dark period. Many studies have demonstrated that AD mouse models expressing sleep deterioration over time tend to exhibit the majority of their changes in sleep behavior during dark (active) period (Jyoti et al., 2010; Colby-Milley et al., 2015; Sethi et al., 2015; Van Erum et al., 2019; Holton et al., 2020; interestingly, the opposite occurred for Maezono et al., 2020). Therefore, research focusing on sleep behavior exclusively during the light period may not provide reliable representation of that model’s general sleep behavior. Furthermore, sleep pressure reduces as time asleep increases making the first hours of the sleep cycle more likely to contain deep sleep and fewer interruptions than the hours immediately preceding the dark period.

#### Varying range designations for frequency power bands

Vigilance states are identified based on the dominant EEG frequency bands, thereby making changes in power spectra a useful tool for diagnosing neurodegeneration (Jeong, 2004). EEG of individuals with AD, for example, have been shown to produce a power spectral shift towards lower frequencies, which is believed to be a consequence of neuronal death and axonal pathologies (Jeong, 2004). AD patients are widely reported to exhibit EEG slowing during wakefulness REM sleep (Hassainia et al., 1994; D'Atri et al., 2021). For AD mouse studies, results in power spectral analysis vary among research groups, possibly stemming at least in part by the different ranges designated to define each frequency band.

#### Using a wide range of ages within a single group of transgenic mice

AD pathology can progress rapidly within a short period of time. Therefore, constructing a single group of mice with widely varying ages (by 1.5 months or more) may result in the combination of mice at different stages of AD pathology. Mice of such widely differing age ranges may exhibit dissimilar sleep patterns.

#### Piezoelectric vs. EEG vs. wireless EEG recording methods

The EEG/EMG combination is a well-established technique for sleep analysis because it can differentiate between all 3 vigilance states identified in mice, detect NREM markers such as K-complexes and sleep spindles, detect pathological markers such as interictal spikes, and can be utilized to generate power spectral data. However, EEG/EMG requires highly invasive surgery and the mouse must be tethered throughout the duration of the recording, which may influence sleep behaviors. While the EEG/EMG system for remains the gold standard for sleep scoring (Bastianini et al., 2017), the piezoelectric system offers many noteworthy advantages in sleep research, namely, the piezoelectric system is a non-invasive technique to analyze sleep that capitalizes on changes in breathing patterns during different vigilance states (Yaghouby et al., 2016). By enabling free movement of the mice under investigation, the piezoelectric approach does not apply discomfort cause by tethering. Additionally, mice would not require EEG implantation surgery and therefore would not suffer operational trauma or potentially unnoticed physical injury from surgery. However, because the piezoelectric system relies on breathing patterns to distinguish between sleep states, it is limited in its capabilities of differentiating between NREM and REM sleep. Wireless EEG devices aim to improve upon the invasiveness and restrictions of traditional EEG equipment. As stated by Jyoti et al. (2015), “Most previous EEG studies in rodents used cabled devices, and vigilance staging relied on EMG, which may not be ideal and equally informative for all age groups. Such experimental parameters may explain the differences compared with our data, as our recording conditions were wireless with activity assessments based on an accelerometer” (Jyoti et al., 2015).

#### Combining male and female mice into a single group

While females are at greater risk of developing AD in humans than males (Turner, 2001), mouse model research is more commonly conducted on male mice in efforts to reduce the effects of confounding variables. Some studies comparing male and female AD mice have demonstrated sexual differences in sleep behavior, either in duration or power spectra (Wisor et al., 2005; Sethi et al., 2015).

Methodological variation is an important component of sleep research. However, a standardized system of measurement is necessary for the reproducibility and comparability of scientific results. Therefore, the implementation of standardized frequency band ranges and epoch definitions may help improve the quality and consistency of sleep studies. Shorter epochs reflect transitions between sleep states with greater accuracy. Furthermore, factors such as the mouse model genetic background, sex and age, as well as the sleep recording system, and recording period should be taken into account when designing or interpreting sleep research using transgenic mouse models of AD. Reducing the age variation within individual groups of mice may reduce the likelihood of a group containing mice at different stages of the disease.

## Conclusion and future directions

AD mouse models exhibit the potential to reproduce a wide range of sleep dysfunctions observed in AD. The most frequently observed sleep impairments in AD mice are reductions in NREM and REM sleep duration. The reduction of REM speed duration, specifically, is the most consistent form of sleep deterioration in AD mice. Interestingly, many mouse models demonstrate hyperactivity during dark phase, reminiscent of Sundown syndrome observed in AD patients. Of the mouse studies that provide data on vigilance state durations and bout lengths, changes in durations either precede or coincide with changes in bout lengths at least one vigilance state (not necessarily the same one). Generally, mice display fewer wake, NREM sleep and REM sleep bouts with the progression of AD pathology. REM slowing (the decreasing of delta and theta frequencies along with increasing of alpha and beta frequencies) is commonly identified in mouse models of AD. Nevertheless, no trend in altered power spectra can be determined consistent across all models or frequencies, likely due to the variation in mouse pathologies and experimental parameters.

Decoding the links (likely more than one) between AD and sleep deterioration presents a multi-variable challenge. Soluble and insoluble Aβ, hyperphosphorylated tau and NFTs, and sex- and age-associated factors may each contribute, separately or in conjunction, to the mechanisms underlying sleep impairment. Plausible mechanisms include the inhibition or destruction sleep-regulating neurons, such as the VLPO, medial septum or the cholinergic neurons of the LDT/PPT. Damage to the brainstem or medial septum by the formation of plaques or synaptic interference by the accumulation of soluble Aβ may be responsible for the observed reductions in REM sleep, as well as associated cognitive declines.

Given the growing population of patients with dementia and the potential for sleep augmentation to decelerate the progression of AD, detailed studies examining the chronology of sleep impairments in individuals with AD are needed. Such studies would provide a road map to which mouse models best emulate specific stages of AD. This knowledge may guide more fruitful investigations into mechanisms elucidating which AD biomarkers of AD would be best targeted for future therapies.

## Author contributions

VJD contributed to the original conception and design of the manuscript, constructed all tables and figures, as well as compiled and organized the data necessary to construct the tables. CW and TK revised and made significant contributions to the content and formatting of the manuscript. All authors contributed to the article and approved the submitted version.

## Funding

This work was supported by Ministry of Health & Welfare (HI22C0467 and HU22C0150 to TK) and Ministry of Science & ICT (NRF-2022R1A2C3009749 to TK).

## Conflict of interest

The authors declare that the research was conducted in the absence of any commercial or financial relationships that could be construed as a potential conflict of interest.

## Publisher’s note

All claims expressed in this article are solely those of the authors and do not necessarily represent those of their affiliated organizations, or those of the publisher, the editors and the reviewers. Any product that may be evaluated in this article, or claim that may be made by its manufacturer, is not guaranteed or endorsed by the publisher.

## Glossary

**Table:** 

A β	Amyloid beta
AD	Alzheimer’s disease
APP	Amyloid precursor protein
EEG	Electroencephalogram
EMG	Electromyogram
NFT	Neurofibrillary tangle
LDT	Laterodorsal tegmentum
LTP	Long-term potentiation
MAPT	Microtubule-associated protein tau
MWM	Morris water maze
nNOS	Neuronal nitric oxide synthase
NREM	Non-rapid eye movement
PS1 or PSEN1 (or PS1)	Presenilin 1
PPT	Pedunculopontine tegmentum
REM	Rapid eye movement
RWM	Radial water maze
SWS	Slow wave sleep
VLPO	Ventrolateral preoptic area
ZT	Zeitgeber
