# Supplementary material for: Progressive sleep disturbance in various transgenic mouse models of Alzheimer’s disease
Source: Front Aging Neurosci. 2023 May 19;15:1119810. doi: 10.3389/fnagi.2023.1119810 (PMC10235623; doi:10.3389/fnagi.2023.1119810)
Supplement: Supplementary file 1 [file Table_1.DOCX]

Supplementary table 1: Chronology of pathologies in various mouse models of Alzheimer’s disease

| **Mouse line** | **Genetics** | **General pathologies (months)** | **Sleep impairment (months)** | **References** |
| --- | --- | --- | --- | --- |
|  | | | | |
| **APP-BASED MOUSE MODELS OF AD** | | | | |
|  | | | | |
| APP23 | - APP mutation: Swedish | - Memory deficits: (3 m) - Plaques: (6 m) - Gliosis: (6 m) - Hyperphosphorylated tau: (6 m) - Neuronal loss (14-18 m) - No synaptic loss - No change in long-term potentiation - No tangles | - Altered power spectra (6 m) - Altered NREM duration (12 m) - Altered REM duration (12 m) | (Boncristiano et al., 2005; Calhoun et al., 1998; Roder et al., 2003; Stalder et al., 1999; Sturchler-Pierrat et al., 1997; Van Dam et al., 2003; Van Erum et al., 2019) |
|  | | | | |
| App^NL-G-F/NL-G-F^ | - APP mutation: Swedish, Iberian, Arctic | - Plaques: (2-5 m) - Gliosis (2-7 m) - Memory deficits: (6 m) - No tau hyperphosphorylation - No tangles - No neurodegeneration | - Altered power spectra (6 m) - Altered NREM count (6 m) - Altered REM duration (6 m) - Altered NREM duration (12 m) - Altered REM length (12 m) | (Castillo et al., 2017; Maezono et al., 2020; Nilsson et al., 2014; Saito et al., 2014) |
|  | | | | |
| J20 | - APP mutation: Swedish, Indiana | - Puncta Aβ (1 m) - Synaptic loss: (3 m) - Neuronal differentiation: (3 m) - Neuronal loss (3 m) - Gliosis (3 m) - Memory deficits: (3-4 m) - Plaques (7 m) - No neuronal loss - No tangles | - Altered power spectra (11-12 m) - Partially altered REM duration (11-12 m) | (Cheng et al., 2007; Filon et al., 2020; Hong et al., 2016; López-Toledano and Shelanski, 2007; Wright et al., 2013) |
|  | | | | |
| PDAPP | - APP mutation: Indiana | - Memory deficits: (3 m) - Reduced long-term potentiation (4-5 m) - Plaques (6-9 m) - Gliosis (6-9 m) - Dendritic loss (2 m) - Synaptic loss (8 m) | - Partially altered REM duration (3-5 m) - Partially altered NREM duration (20-26 m) - Altered REM length (20-26 m) - Altered NREM length (20-26 m) - Altered REM count (20-26 m) | (Dodart et al., 1999; Games et al., 1995; Huitrón-Reséndiz et al., 2002; Lanz et al., 2003; Larson et al., 1999) |
|  | | | | |
| Tg2576 | - APP mutation: Swedish | - Synaptic loss (2-4.5 m) - Reduced long-term potentiation (6 m) - Memory deficits (6 m) - Plaques: (9-18 m) - Gliosis (10-16 m) | - Interictal spikes (1.5 m) - Altered REM duration (6 m) - Altered REM length (6 m) - Altered power spectra (8-12 m) | (Frautschy et al., 1998; Hsiao et al., 1996; Irizarry et al., 1997; Jacobsen et al., 2006; Kam et al., 2016; Kent et al., 2018; Lanz et al., 2003; Wisor et al., 2005; Zhang et al., 2005) |
|  | | | | |
| TgCRND8 | - APP mutation: Swedish, Indiana | - Plaques (2-3 m) - Memory deficits (3 m) - Gliosis (3-3.5 m) - Reduced long-term potentiation (6 m) - Synaptic loss (6 m) - Neuronal loss (6 m) - Hyperphosphorylated tau (7 m) | - Altered power spectra (3 m) - Altered NREM duration (3 m) - Altered REM duration (3 m) | (Adalbert et al., 2009; Bellucci et al., 2007; Brautigam et al., 2012; Chishti et al., 2001; Colby-Milley et al., 2015; Dudal et al., 2004; Kimura et al., 2012) |
|  | | | | |
| **APP AND PSEN DOUBLE TRANSGENIC MOUSE MODELS OF AD** | | | | |
|  | | | | |
| 5XFAD | - APP mutations: Swedish, London, Florida - PSEN1 mutation: PSEN1, L286V | - Plaques (1.5 m) - Gliosis (2 m) - Memory deficits (4-5 m) - Synaptic loss (4 m) - Reduced long-term potentiation (6 m) - Neuronal loss (9 m) (ALZFORUM claims earlier) | - Altered total sleep duration (4-4.5 m) - Altered total sleep length (4-4.5 m) - Altered power spectra (6 m) | (Buskila et al., 2013; Duncan et al., 2019; Eimer and Vassar, 2013; Kimura and Ohno, 2009; Oakley et al., 2006; Schneider et al., 2014; Sethi et al., 2015) |
|  | | | | |
| AβPP^swe^/PS1^∆E9^ | - APP mutation: Swedish - PSEN1: ΔE9 | - Long-term potentiation (3 m) - Synaptic loss (4 m) - Plaques (6 m) - Gliosis (6 m) - Memory deficits (6-12 m) | - Altered power spectra (3 m) - Altered NREM duration (3 m) - Altered REM duration (9 m) | (Hong et al., 2016; Jankowsky et al., 2004; Kamphuis et al., 2012; Malm et al., 2011; Roh et al., 2012; Volianskis et al., 2010; Zhang et al., 2019) |
|  | | | | |
| AβPP^swe^/PS1^A246E^ | - APP mutation: Swedish - PSEN1 mutation: A246E | - Memory deficits (3 m) - Plaques (6-9 m) - Neuronal loss (10 m) | - Altered NREM duration (5 m) | (Casas et al., 2004; Dewachter et al., 2000; Filali and Lalonde, 2015; Jyoti et al., 2010; Liu et al., 2002) |
|  | | | | |
| **APP. PSEN AND TAU TRANSGENIC MOUSE MODELS OF AD** | | | | |
|  | | | | |
| 3xTgAD | - APP mutation: Swedish - PSEN1 mutation: M146V - Tau mutation: MAPT P301L | - Intracellular Aβ (2 m) - Gliosis (2 m) - Tau pathology (2 m) - Memory deficits (4 m) - Plaques (6 m) - Reduced long-term potentiation (6 m) | - No changes to sleep duration (18 m) | (Billings et al., 2005; Kent et al., 2018; Mastrangelo and Bowers, 2008; Oddo et al., 2003) |
|  | | | | |
| PLB1 | - APP mutation: Swedish, London - PSEN1 mutation: A246E - Tau mutation: MAPT P301L, R406W | - Memory deficits (4 m) - Tau pathology (6 m) - Reduced long-term potentiation (6 m) - Few plaques (6-13 m) - Synaptic dysfunction (12 m) | - Altered power spectra (5 m) - Altered NREM duration (5 m) - Altered REM duration (13 m) | (Jyoti et al., 2015; Koss et al., 2013; Platt et al., 2011; Ryan et al., 2013) |
|  | | | | |
| **OTHER MOUSE MODELS OF AD** | | | | |
|  | | | | |
| APPSwDI/NOS2 -/- (CVN-AD) | - APP mutations: Swedish, Iowa, E693Q | - Plaques (3 m) - Neuronal loss (5.5-12 m) - Gliosis (8-9 m) - Memory deficits (12 m) - Tau pathology (12 m) | - Somewhat altered NREM duration (8-9 m) - Altered total sleep count (8-9 m) | (Nwafor et al., 2021; Turner, 2021; Wilcock et al., 2008) |
|  | | | | |
| P301S Tau | - Tau mutation: MAPT P301S | - Gliosis (3 m) - Tau pathology (4 m) - Tangles (5 m) - Synaptic loss (6 m) - Reduced long-term potentiation (6 m) - Memory impairment (6 m) - Neuronal loss (8 m) | - Altered power spectra (6 m) - Altered REM duration (9 m) - Altered NREM duration (11 m) - Altered NREM count (9 m) - Altered REM count (9 m) - Altered NREM length (9 m) - Altered REM length (11 m) | (Holth et al., 2017; Takeuchi et al., 2011; Yoshiyama et al., 2007) |
|  | | | | |
| rTg4510 | - Tau mutation: MAPT P301L | - Tau pathology (2-2.5 m) - Memory impairment (2-2.5 m) - Gliosis (2.5 m) - Reduced long-term potentiation (4.5 m) - Neuronal loss (5.5-8.5 m) - Synaptic loss (7.5 m) | - Altered power spectra (4.5 m) - Altered REM length (5.5 m) - Altered REM count (5.5 m) - Altered NREM duration (6.5 m) | (Helboe et al., 2017; Holton et al., 2020; Hoover et al., 2010; Kopeikina et al., 2013; Ramsden et al., 2005; Santacruz et al., 2005; Wes et al., 2014) |
|  | | | | |
| SAMP8 | - Spontaneous | - Gliosis (1-2 m) - Memory impairment (2 m) - Neuronal loss (2 m) - Plaques (6 m) - Reduced long-term potentiation (12 m) | - Altered NREM duration (4 m) - Altered REM duration (4 m) | (Armbrecht et al., 2014; Beuckmann et al., 2021; Del Valle et al., 2010; Kawamata et al., 1997; Miyamoto et al., 1986) |

**References**

Adalbert, R., Nogradi, A., Babetto, E., Janeckova, L., Walker, S. A., Kerschensteiner, M., et al. (2009). Severely dystrophic axons at amyloid plaques remain continuous and connected to viable cell bodies. *Brain* 132, 402-416.

Armbrecht, H. J., Siddiqui, A. M., Green, M., Farr, S. A., Kumar, V. B., Banks, W. A., et al. (2014). SAMP8 mice have altered hippocampal gene expression in long term potentiation, phosphatidylinositol signaling, and endocytosis pathways. *Neurobiol. Aging* 35, 159-168.

Bellucci, A., Rosi, M. C., Grossi, C., Fiorentini, A., Luccarini, I., and Casamenti, F. (2007). Abnormal processing of tau in the brain of aged TgCRND8 mice. *Neurobiol. Dis.* 27, 328-338.

Beuckmann, C. T., Suzuki, H., Musiek, E. S., Ueno, T., Sato, T., Bando, M., et al. (2021). Evaluation of SAMP8 Mice as a Model for Sleep-Wake and Rhythm Disturbances Associated with Alzheimer’s Disease: Impact of Treatment with the Dual Orexin (Hypocretin) Receptor Antagonist Lemborexant. *J. Alzheimers Dis.*, 1-16.

Billings, L. M., Oddo, S., Green, K. N., McGaugh, J. L., and LaFerla, F. M. (2005). Intraneuronal Aβ causes the onset of early Alzheimer’s disease-related cognitive deficits in transgenic mice. *Neuron* 45, 675-688.

Boncristiano, S., Calhoun, M. E., Howard, V., Bondolfi, L., Kaeser, S. A., Wiederhold, K.-H., et al. (2005). Neocortical synaptic bouton number is maintained despite robust amyloid deposition in APP23 transgenic mice. *Neurobiol. Aging* 26, 607-613.

Brautigam, H., Steele, J. W., Westaway, D., Fraser, P. E., St George-Hyslop, P. H., Gandy, S., et al. (2012). The isotropic fractionator provides evidence for differential loss of hippocampal neurons in two mouse models of Alzheimer's disease. *Mol. Neurodegener.* 7, 1-5.

Buskila, Y., Crowe, S. E., and Ellis-Davies, G. C. (2013). Synaptic deficits in layer 5 neurons precede overt structural decay in 5xFAD mice. *Neuroscience* 254, 152-159.

Calhoun, M. E., Wiederhold, K.-H., Abramowski, D., Phinney, A. L., Probst, A., Sturchler-Pierrat, C., et al. (1998). Neuron loss in APP transgenic mice. *Nature* 395, 755-756.

Casas, C., Sergeant, N., Itier, J.-M., Blanchard, V., Wirths, O., Van Der Kolk, N., et al. (2004). Massive CA1/2 neuronal loss with intraneuronal and N-terminal truncated Aβ42 accumulation in a novel Alzheimer transgenic model. *The American journal of pathology* 165, 1289-1300.

Castillo, E., Leon, J., Mazzei, G., Abolhassani, N., Haruyama, N., Saito, T., et al. (2017). Comparative profiling of cortical gene expression in Alzheimer’s disease patients and mouse models demonstrates a link between amyloidosis and neuroinflammation. *Sci. Rep.* 7, 1-16.

Cheng, I. H., Scearce-Levie, K., Legleiter, J., Palop, J. J., Gerstein, H., Bien-Ly, N., et al. (2007). Accelerating amyloid-β fibrillization reduces oligomer levels and functional deficits in Alzheimer disease mouse models. *J. Biol. Chem.* 282, 23818-23828.

Chishti, M. A., Yang, D.-S., Janus, C., Phinney, A. L., Horne, P., Pearson, J., et al. (2001). Early-onset amyloid deposition and cognitive deficits in transgenic mice expressing a double mutant form of amyloid precursor protein 695. *J. Biol. Chem.* 276, 21562-21570.

Colby-Milley, J., Cavanagh, C., Jego, S., Breitner, J. C., Quirion, R., and Adamantidis, A. (2015). Sleep-wake cycle dysfunction in the TgCRND8 mouse model of Alzheimer’s disease: from early to advanced pathological stages. *PLoS One* 10, e0130177.

Del Valle, J., Duran-Vilaregut, J., Manich, G., Casadesús, G., Smith, M. A., Camins, A., et al. (2010). Early amyloid accumulation in the hippocampus of SAMP8 mice. *J. Alzheimers Dis.* 19, 1303-1315.

Dewachter, I., Van Dorpe, J., Smeijers, L., Gilis, M., Kuipéri, C., Laenen, I., et al. (2000). Aging increased amyloid peptide and caused amyloid plaques in brain of old APP/V717I transgenic mice by a different mechanism than mutant presenilin1. *J. Neurosci.* 20, 6452-6458.

Dodart, J.-C., Meziane, H., Mathis, C., Bales, K. R., Paul, S. M., and Ungerer, A. (1999). Behavioral disturbances in transgenic mice overexpressing the V717F Β-amyloid precursor protein. *Behav. Neurosci.* 113, 982.

Dudal, S., Krzywkowski, P., Paquette, J., Morissette, C., Lacombe, D., Tremblay, P., et al. (2004). Inflammation occurs early during the Aβ deposition process in TgCRND8 mice. *Neurobiol. Aging* 25, 861-871.

Duncan, M. J., Farlow, H., Tirumalaraju, C., Yun, D.-H., Wang, C., Howard, J. A., et al. (2019). Effects of the dual orexin receptor antagonist DORA-22 on sleep in 5XFAD mice. *Alzheimer's & Dementia: Translational Research & Clinical Interventions* 5, 70-80.

Eimer, W. A., and Vassar, R. (2013). Neuron loss in the 5XFAD mouse model of Alzheimer’s disease correlates with intraneuronal Aβ42 accumulation and Caspase-3 activation. *Mol. Neurodegener.* 8, 1-12.

Filali, M., and Lalonde, R. (2015). Motor activity in young APPswe+ PS1/A246E bigenic mice as a predicting variable for memory decline. *J. Neurosci. Res.* 93, 948-953.

Filon, M. J., Wallace, E., Wright, S., Douglas, D. J., Steinberg, L. I., Verkuilen, C. L., et al. (2020). Sleep and diurnal rest-activity rhythm disturbances in a mouse model of Alzheimer’s disease. *Sleep* 43, zsaa087.

Frautschy, S. A., Yang, F., Irrizarry, M., Hyman, B., Saido, T., Hsiao, K., et al. (1998). Microglial response to amyloid plaques in APPsw transgenic mice. *The American journal of pathology* 152, 307.

Games, D., Adams, D., Alessandrini, R., Barbour, R., Borthelette, P., Blackwell, C., et al. (1995). Alzheimer-type neuropathology in transgenic mice overexpressing V717F β-amyloid precursor protein. *Nature* 373, 523-527.

Helboe, L., Egebjerg, J., Barkholt, P., and Volbracht, C. (2017). Early depletion of CA1 neurons and late neurodegeneration in a mouse tauopathy model. *Brain Res.* 1665, 22-35.

Holth, J. K., Mahan, T. E., Robinson, G. O., Rocha, A., and Holtzman, D. M. (2017). Altered sleep and EEG power in the P301S Tau transgenic mouse model. *Annals of clinical and translational neurology* 4, 180-190.

Holton, C., Hanley, N., Shanks, E., Oxley, P., McCarthy, A., Eastwood, B. J., et al. (2020). Longitudinal changes in EEG power, sleep cycles and behaviour in a tau model of neurodegeneration. *Alzheimers Res. Ther.* 12, 1-15.

Hong, S., Beja-Glasser, V. F., Nfonoyim, B. M., Frouin, A., Li, S., Ramakrishnan, S., et al. (2016). Complement and microglia mediate early synapse loss in Alzheimer mouse models. *Science* 352, 712-716.

Hoover, B. R., Reed, M. N., Su, J., Penrod, R. D., Kotilinek, L. A., Grant, M. K., et al. (2010). Tau mislocalization to dendritic spines mediates synaptic dysfunction independently of neurodegeneration. *Neuron* 68, 1067-1081.

Hsiao, K., Chapman, P., Nilsen, S., Eckman, C., Harigaya, Y., Younkin, S., et al. (1996). Correlative memory deficits, Aβ elevation, and amyloid plaques in transgenic mice. *Science* 274, 99-103.

Huitrón-Reséndiz, S., Sánchez-Alavez, M., Gallegos, R., Berg, G., Crawford, E., Giacchino, J. L., et al. (2002). Age-independent and age-related deficits in visuospatial learning, sleep–wake states, thermoregulation and motor activity in PDAPP mice. *Brain Res.* 928, 126-137.

Irizarry, M. C., McNamara, M., Fedorchak, K., Hsiao, K., and Hyman, B. T. (1997). APPSw transgenic mice develop age-related Aβ deposits and neuropil abnormalities, but no neuronal loss in CA1. *J. Neuropathol. Exp. Neurol.* 56, 965-973.

Jacobsen, J. S., Wu, C.-C., Redwine, J. M., Comery, T. A., Arias, R., Bowlby, M., et al. (2006). Early-onset behavioral and synaptic deficits in a mouse model of Alzheimer's disease. *Proceedings of the National Academy of Sciences* 103, 5161-5166.

Jankowsky, J. L., Fadale, D. J., Anderson, J., Xu, G. M., Gonzales, V., Jenkins, N. A., et al. (2004). Mutant presenilins specifically elevate the levels of the 42 residue β-amyloid peptide in vivo: evidence for augmentation of a 42-specific γ secretase. *Hum. Mol. Genet.* 13, 159-170.

Jyoti, A., Plano, A., Riedel, G., and Platt, B. (2010). EEG, Activity, and Sleep Architecture in a Transgenic AβPP swe/PSEN1 A246E Alzheimer's Disease Mouse. *J. Alzheimers Dis.* 22, 873-887.

---. (2015). Progressive age-related changes in sleep and EEG profiles in the PLB1Triple mouse model of Alzheimer’s disease. *Neurobiol. Aging* 36, 2768-2784.

Kam, K., Duffy, Á. M., Moretto, J., LaFrancois, J. J., and Scharfman, H. E. (2016). Interictal spikes during sleep are an early defect in the Tg2576 mouse model of β-amyloid neuropathology. *Sci. Rep.* 6, 1-16.

Kamphuis, W., Mamber, C., Moeton, M., Kooijman, L., Sluijs, J. A., Jansen, A. H., et al. (2012). GFAP isoforms in adult mouse brain with a focus on neurogenic astrocytes and reactive astrogliosis in mouse models of Alzheimer disease.

Kawamata, T., Akiguchi, I., Yagi, H., Irino, M., Sugiyama, H., Akiyama, H., et al. (1997). Neuropathological studies on strains of senescence-accelerated mice (SAM) with age-related deficits in learning and memory. *Exp. Gerontol.* 32, 161-169.

Kent, B. A., Strittmatter, S. M., and Nygaard, H. B. (2018). Sleep and EEG power spectral analysis in three transgenic mouse models of Alzheimer’s disease: APP/PS1, 3xTgAD, and Tg2576. *J. Alzheimers Dis.* 64, 1325-1336.

Kimura, R., MacTavish, D., Yang, J., Westaway, D., and Jhamandas, J. H. (2012). Beta amyloid-induced depression of hippocampal long-term potentiation is mediated through the amylin receptor. *J. Neurosci.* 32, 17401-17406.

Kimura, R., and Ohno, M. (2009). Impairments in remote memory stabilization precede hippocampal synaptic and cognitive failures in 5XFAD Alzheimer mouse model. *Neurobiol. Dis.* 33, 229-235.

Kopeikina, K. J., Polydoro, M., Tai, H. C., Yaeger, E., Carlson, G. A., Pitstick, R., et al. (2013). Synaptic alterations in the rTg4510 mouse model of tauopathy. *J. Comp. Neurol.* 521, 1334-1353.

Koss, D. J., Drever, B. D., Stoppelkamp, S., Riedel, G., and Platt, B. (2013). Age-dependent changes in hippocampal synaptic transmission and plasticity in the PLB1Triple Alzheimer mouse. *Cell. Mol. Life Sci.* 70, 2585-2601.

Lanz, T., Carter, D., and Merchant, K. (2003). Dendritic spine loss in the hippocampus of young PDAPP and Tg2576 mice and its prevention by the ApoE2 genotype. *Neurobiol. Dis.* 13, 246-253.

Larson, J., Lynch, G., Games, D., and Seubert, P. (1999). Alterations in synaptic transmission and long-term potentiation in hippocampal slices from young and aged PDAPP mice. *Brain Res.* 840, 23-35.

Liu, L., Ikonen, S., Heikkinen, T., Heikkilä, M., Puoliväli, J., van Groen, T., et al. (2002). Effects of fimbria-fornix lesion and amyloid pathology on spatial learning and memory in transgenic APP+ PS1 mice. *Behav. Brain Res.* 134, 433-445.

López-Toledano, M. A., and Shelanski, M. L. (2007). Increased Neurogenesis in Young Transgenic Mice Overexpressing Human APP~ S~ w~,~~ I~ n~ d. *J. Alzheimers Dis.* 12, 229-240.

Maezono, S. E. B., Kanuka, M., Tatsuzawa, C., Morita, M., Kawano, T., Kashiwagi, M., et al. (2020). Progressive changes in sleep and its relations to amyloid-β distribution and learning in single App knock-in mice. *Eneuro* 7.

Malm, T., Koistinaho, J., and Kanninen, K. (2011). Utilization of APPswe/PS1dE9 transgenic mice in research of Alzheimer's disease: focus on gene therapy and cell-based therapy applications. *International journal of Alzheimer’s disease* 2011.

Mastrangelo, M. A., and Bowers, W. J. (2008). Detailed immunohistochemical characterization of temporal and spatial progression of Alzheimer's disease-related pathologies in male triple-transgenic mice. *BMC Neurosci.* 9, 1-31.

Miyamoto, M., Kiyota, Y., Yamazaki, N., Nagaoka, A., Matsuo, T., Nagawa, Y., et al. (1986). Age-related changes in learning and memory in the senescence-accelerated mouse (SAM). *Physiol. Behav.* 38, 399-406.

Nilsson, P., Saito, T., and Saido, T. C. 2014. New mouse model of Alzheimer’s. Pages 499-502: ACS Publications.

Nwafor, D. C., Chakraborty, S., Jun, S., Brichacek, A. L., Dransfeld, M., Gemoets, D. E., et al. (2021). Disruption of metabolic, sleep, and sensorimotor functional outcomes in a female transgenic mouse model of Alzheimer’s disease. *Behav. Brain Res.* 398, 112983.

Oakley, H., Cole, S. L., Logan, S., Maus, E., Shao, P., Craft, J., et al. (2006). Intraneuronal β-amyloid aggregates, neurodegeneration, and neuron loss in transgenic mice with five familial Alzheimer's disease mutations: potential factors in amyloid plaque formation. *J. Neurosci.* 26, 10129-10140.

Oddo, S., Caccamo, A., Shepherd, J. D., Murphy, M. P., Golde, T. E., Kayed, R., et al. (2003). Triple-transgenic model of Alzheimer's disease with plaques and tangles: intracellular Aβ and synaptic dysfunction. *Neuron* 39, 409-421.

Platt, B., Drever, B., Koss, D., Stoppelkamp, S., Jyoti, A., Plano, A., et al. (2011). Abnormal cognition, sleep, EEG and brain metabolism in a novel knock-in Alzheimer mouse, PLB1. *PLoS One* 6, e27068.

Ramsden, M., Kotilinek, L., Forster, C., Paulson, J., McGowan, E., SantaCruz, K., et al. (2005). Age-dependent neurofibrillary tangle formation, neuron loss, and memory impairment in a mouse model of human tauopathy (P301L). *J. Neurosci.* 25, 10637-10647.

Roder, S., Danober, L., Pozza, M., Lingenhoehl, K., Wiederhold, K.-H., and Olpe, H.-R. (2003). Electrophysiological studies on the hippocampus and prefrontal cortex assessing the effects of amyloidosis in amyloid precursor protein 23 transgenic mice. *Neuroscience* 120, 705-720.

Roh, J. H., Huang, Y., Bero, A. W., Kasten, T., Stewart, F. R., Bateman, R. J., et al. (2012). Disruption of the sleep-wake cycle and diurnal fluctuation of β-amyloid in mice with Alzheimer’s disease pathology. *Sci. Transl. Med.* 4, 150ra122-150ra122.

Ryan, D., Koss, D., Porcu, E., Woodcock, H., Robinson, L., Platt, B., et al. (2013). Spatial learning impairments in PLB1 triple knock-in Alzheimer mice are task-specific and age-dependent. *Cell. Mol. Life Sci.* 70, 2603-2619.

Saito, T., Matsuba, Y., Mihira, N., Takano, J., Nilsson, P., Itohara, S., et al. (2014). Single App knock-in mouse models of Alzheimer's disease. *Nat. Neurosci.* 17, 661-663.

Santacruz, K., Lewis, J., Spires, T., Paulson, J., Kotilinek, L., Ingelsson, M., et al. (2005). Tau suppression in a neurodegenerative mouse model improves memory function. *Science* 309, 476-481.

Schneider, F., Baldauf, K., Wetzel, W., and Reymann, K. (2014). Behavioral and EEG changes in male 5xFAD mice. *Physiol. Behav.* 135, 25-33.

Sethi, M., Joshi, S. S., Webb, R. L., Beckett, T. L., Donohue, K. D., Murphy, M. P., et al. (2015). Increased fragmentation of sleep–wake cycles in the 5XFAD mouse model of Alzheimer’s disease. *Neuroscience* 290, 80-89.

Stalder, M., Phinney, A., Probst, A., Sommer, B., Staufenbiel, M., and Jucker, M. (1999). Association of microglia with amyloid plaques in brains of APP23 transgenic mice. *The American journal of pathology* 154, 1673-1684.

Sturchler-Pierrat, C., Abramowski, D., Duke, M., Wiederhold, K.-H., Mistl, C., Rothacher, S., et al. (1997). Two amyloid precursor protein transgenic mouse models with Alzheimer disease-like pathology. *Proceedings of the National Academy of Sciences* 94, 13287-13292.

Takeuchi, H., Iba, M., Inoue, H., Higuchi, M., Takao, K., Tsukita, K., et al. (2011). P301S mutant human tau transgenic mice manifest early symptoms of human tauopathies with dementia and altered sensorimotor gating. *PLoS One* 6, e21050.

Turner, D. A. (2021). Contrasting metabolic insufficiency in aging and dementia. *Aging Dis.* 12, 1081.

Van Dam, D., d'Hooge, R., Staufenbiel, M., Van Ginneken, C., Van Meir, F., and De Deyn, P. P. (2003). Age‐dependent cognitive decline in the APP23 model precedes amyloid deposition. *Eur. J. Neurosci.* 17, 388-396.

Van Erum, J., Van Dam, D., Sheorajpanday, R., and De Deyn, P. P. (2019). Sleep architecture changes in the APP23 mouse model manifest at onset of cognitive deficits. *Behav. Brain Res.* 373, 112089.

Volianskis, A., Køstner, R., Mølgaard, M., Hass, S., and Jensen, M. S. (2010). Episodic memory deficits are not related to altered glutamatergic synaptic transmission and plasticity in the CA1 hippocampus of the APPswe/PS1ΔE9-deleted transgenic mice model of β-amyloidosis. *Neurobiol. Aging* 31, 1173-1187.

Wes, P. D., Easton, A., Corradi, J., Barten, D. M., Devidze, N., DeCarr, L. B., et al. (2014). Tau overexpression impacts a neuroinflammation gene expression network perturbed in Alzheimer’s disease. *PLoS One* 9, e106050.

Wilcock, D. M., Lewis, M. R., Van Nostrand, W. E., Davis, J., Previti, M. L., Gharkholonarehe, N., et al. (2008). Progression of amyloid pathology to Alzheimer's disease pathology in an amyloid precursor protein transgenic mouse model by removal of nitric oxide synthase 2. *J. Neurosci.* 28, 1537-1545.

Wisor, J., Edgar, D., Yesavage, J., Ryan, H., McCormick, C., Lapustea, N., et al. (2005). Sleep and circadian abnormalities in a transgenic mouse model of Alzheimer’s disease: a role for cholinergic transmission. *Neuroscience* 131, 375-385.

Wright, A. L., Zinn, R., Hohensinn, B., Konen, L. M., Beynon, S. B., Tan, R. P., et al. (2013). Neuroinflammation and neuronal loss precede Aβ plaque deposition in the hAPP-J20 mouse model of Alzheimer’s disease. *PLoS One* 8, e59586.

Yoshiyama, Y., Higuchi, M., Zhang, B., Huang, S.-M., Iwata, N., Saido, T. C., et al. (2007). Synapse loss and microglial activation precede tangles in a P301S tauopathy mouse model. *Neuron* 53, 337-351.

Zhang, B., Veasey, S. C., Wood, M. A., Leng, L. Z., Kaminski, C., Leight, S., et al. (2005). Impaired rapid eye movement sleep in the Tg2576 APP murine model of Alzheimer's disease with injury to pedunculopontine cholinergic neurons. *The American journal of pathology* 167, 1361-1369.

Zhang, F., Zhong, R., Li, S., Fu, Z., Wang, R., Wang, T., et al. (2019). Alteration in sleep architecture and electroencephalogram as an early sign of Alzheimer's disease preceding the disease pathology and cognitive decline. *Alzheimer's & Dementia* 15, 590-597.
