# Supplementary material for: Progressive sleep disturbance in various transgenic mouse models of Alzheimer’s disease
Source: Front Aging Neurosci. 2023 May 19;15:1119810. doi: 10.3389/fnagi.2023.1119810 (PMC10235623; doi:10.3389/fnagi.2023.1119810)
Supplement: Supplementary file 2 [file Table_2.DOCX]

Supplementary table 2: Changes in vigilance state durations in various mouse models of Alzheimer’s disease compared to age-matched controls

| **Mouse line** | **Sex** | **Age (months)** | **Wake** | **NREM sleep** | **REM sleep** | | **Total sleep** | | **REM/**  **T.S.** | **System** | **Units (period)** | **References** |
| --- | --- | --- | --- | --- | --- | --- | --- | --- | --- | --- | --- | --- |
|  | | | | | | | | | | | | |
| **APP-BASED MOUSE MODELS OF AD** | | | | | | | | | | | | |
|  | | | | | | | | | | | | |
| APP23 | M | 3  6  12 | ↑  ns  ns  ns  ns  ns  ↑  ns  ↑ | ns  ns  ns  ns  ns  ns  ↓  ns  ↓ | ns  ns  ns  ns  ns  ns  ↓  ns  ↓ | | N/A  N/A  N/A  N/A  N/A  N/A  N/A  N/A  N/A | | N/A  N/A  N/A  N/A  N/A  N/A  N/A  N/A  N/A | EEG | sec (24hr)  sec (light)  sec (dark)  sec (24hr)  sec (light)  sec (dark)  sec (24hr)  sec (light)  sec (dark) | (Van Erum et al., 2019) |
|  | | | | | | | | | | | | |
| App^NL-G-F/NL-G-F^ | M | 6  12 | ns  ns  ns  ↑↑  ns  ↑ | ns  ns  ns  ↓  ns  ↓ | ↓  ↓↓  ns  ↓↓↓  ↓↓↓  ns | | N/A  N/A  N/A  N/A  N/A  N/A | | ↓  ↓↓  ns  ↓↓↓  ↓↓↓  ns | EEG | min (24hr)  min (light)  min (dark)  min (24hr)  min (light)  min (dark) | (Maezono et al., 2020) |
|  | | | | | | | | | | | | |
| J20 | Not specified | 11-12 | ns  ns  ns  ns | ns  ns  ns  ns | ns  ↓  ns  ns | | N/A  N/A  N/A  N/A | | N/A  N/A  N/A  N/A | EEG | % (ZT0-6)  % (ZT6-12)  % (ZT12-18)  % (ZT18-24) | (Filon et al., 2020) |
|  | | | | | | | | | | | | |
| PDAPP | F | 3-5  20-26 | ns  ns  ns  ns  ns  ns  ns  ns  ↓  ↓  ↓  ↓  ns  ns  ↑  ↑ | ns  ns  ns  ns  ns  ns  ns  ns  ↑  ↑  ↑  ↑  ns  ns  ↓  ↓ | | ns  ↓  ↓  ns  ns  ns  ns  ns  ns  ns  ns  ns  ns  ↓  ↓  ns | N/A  N/A  N/A  N/A  N/A  N/A  N/A  N/A  N/A  N/A  N/A  N/A  N/A  N/A  N/A  N/A | | N/A  N/A  N/A  N/A  N/A  N/A  N/A  N/A  N/A  N/A  N/A  N/A  N/A  N/A  N/A  N/A | EEG | % (ZT0-3)  % (ZT3-6)  % (ZT6-9)  % (ZT9-12)  % (ZT12-15)  % (ZT15-18)  % (ZT18-21)  % (ZT21-24)  % (ZT0-3)  % (ZT3-6)  % (ZT6-9)  % (ZT9-12)  % (ZT12-15)  % (ZT15-18)  % (ZT18-21)  % (ZT21-24) | (Huitrón-Reséndiz et al., 2002) |
|  | | | | | | | | | | | | |
| Tg2576 | Not specified | 2  6  12 | N/A  N/A  N/A | N/A  N/A  N/A | ns  ↓  ↓ | | N/A  N/A  N/A | | N/A  N/A  N/A | EEG | min (24hr)  min (24hr)  min (24hr) | (Zhang Bin et al., 2005) |
|  | | | | | | | | | | | | |
|  | M      F | 8  11  15  17  22 | N/A  N/A  N/A  N/A  N/A | ns  ns  ns  ns | ns  ns  ns  ns  ↓ | | N/A  N/A  N/A  N/A  N/A | | N/A  N/A  N/A  N/A  N/A | EEG | % (24hr)  % (24hr)  % (24hr)  % (24hr)  % (24hr) | (Wisor et al., 2005) |
|  |  |  |  | N/A |  |  |  |  |  |  |  |  |
|  | | | | | | | | | | | | |
|  | M/F | 12 | ns | ns | ns | | N/A | | N/A | EEG | % (24hr) | (Kent et al., 2018) |
|  | | | | | | | | | | | | |
| TgCRND8 | M | 3  7  11 | ↑  ↑↑↑  ↑  ↑↑↑  ↑↑  ↑↑ | ↓  ↓↓↓  ↓  ↓↓↓  ↓  ↓↓ | ns  ↓↓↓  ns  ↓↓↓  ns  ns | | N/A  N/A  N/A  N/A  N/A  N/A | | N/A  N/A  N/A  N/A  N/A  N/A | EEG | % (light)  % (dark)  % (light)  % (dark)  % (light)  % (dark) | (Colby-Milley et al., 2015) |
|  | | | | | | | | | | | | |
| **APP AND PSEN DOUBLE TRANSGENIC MOUSE MODELS OF AD** | | | | | | | | | | | | |
|  | | | | | | | | | | | | |
| 5XFAD | M/F | 4-4.5 | N/A  N/A  N/A | N/A  N/A  N/A | N/A  N/A  N/A | | ↓↓  ↓  ↓ | | N/A  N/A  N/A | Piezoelectric | % (24hr)  % (light)  % (dark) | (Duncan et al., 2019) |
|  | | | | | | | | | | | | |
|  | M    F | 4-6.5  4-6.5 | N/A  N/A  N/A  N/A  N/A  N/A | N/A  N/A  N/A  N/A  N/A  N/A | N/A  N/A  N/A  N/A  N/A  N/A | | ns  ns  ns  ↓  ns  ↓↓ | | N/A  N/A  N/A  N/A  N/A  N/A | Piezoelectric | % (24hr)  % (light)  % (dark)  % (24hr)  % (light)  % (dark) | (Sethi et al., 2015) |
|  | | | | | | | | | | | | |
|  | M | 9 | ns | ns | ns | | N/A | | ↓ | EEG | % (ZT3-10) | (Schneider et al., 2014) |
|  | | | | | | | | | | | | |
|  | M    F | 10-11  10-11 | ns  ns  ns  ns | ns  ns  ns  ns | ns  ns  ns  ns | | N/A  N/A  N/A  N/A | | N/A  N/A  N/A  N/A | EEG | % (light)  % (dark)  % (light)  % (dark) | (Oblak et al., 2021) |
|  | | | | | | | | | | | | |
| AβPP^swe^/PS1^∆E9^ | Not specified | 3  4  6 | ↑↑  ns  ↑  ns  ↓↓  ↓  ns  ↑↑  ↓ | ↓↓↓  ↓  ↓  ns  ↓↓↓  ↑↑  ns  ↓↓  ↑ | ns  ns  ns  ns  ns  ns  ns  ns  ns | | N/A  N/A  N/A  N/A  N/A  N/A  N/A  N/A  N/A | | N/A  N/A  N/A  N/A  N/A  N/A  N/A  N/A  N/A | EEG | min (24hr)  min (light)  min (dark)  min (24hr)  min (light)  min (dark)  min (24hr)  min (light)  min (dark) | (Zhang Feng et al., 2019) |
|  | | | | | | | | | | | | |
|  | F | 3*  6*  9 | ns  ns  ns  ns  ns  ns  ↑↑↑  ↑↑↑  ↑ | ns  ns  ns  ns  ns  ns  ↓↓  ↓  ↓ | ns  ns  ns  ns  ns  ns  ↓  ↓  ↓ | | N/A  N/A  N/A  N/A  N/A  N/A  N/A  N/A  N/A | | N/A  N/A  N/A  N/A  N/A  N/A  N/A  N/A  N/A | EEG | min (24hr)  min (light)  min (dark)  min (24hr)  min (light)  min (dark)  min (24hr)  min (light)  min (dark) | (Roh et al., 2012) |
|  | | | | | | | | | | | | |
|  | M/F | 8 to 10 | ns | ns | ns | | N/A | | N/A | EEG | % (24hr) | (Kent et al., 2018) |
|  | | | | | | | | | | | | |
|  | F | 12 | ns | ns | ns | | N/A | | N/A | EEG | % (24hr) | (Kent et al., 2019) |
|  | | | | | | | | | | | | |
| AβPP^swe^/PS1^A246E^ | Not specified | 5  20 | ↑↑  ns  ↑↑  ↑↑  ↑  ↑ | ↓  ns  ↓↓↓  ↓  ↓  ↓↓ | ns  ns  ns  ns  ns  ns | | N/A  N/A  N/A  N/A  N/A  N/A | | N/A  N/A  N/A  N/A  N/A  N/A | Wireless EEG | sec (24hr)  sec (light)  sec (dark)  sec (24hr)  sec (light)  sec (dark) | (Jyoti et al., 2010) |
|  | | | | | | | | | | | | |
| **APP. PSEN AND TAU TRANSGENIC MOUSE MODELS OF AD** | | | | | | | | | | | | |
|  | | | | | | | | | | | | |
| 3xTgAD | M/F | 18 | ns | ns | ns | | N/A | | N/A | EEG | % (24hr) | (Kent et al., 2018) |
|  | | | | | | | | | | | | |
| PLB1 | M/F | 5  9  13  17  21 | ns  ns  ns  ns  ↑  ns  ↑  ns  ns  ns | ns  ns  ns  ns  ns  ns  ns  ns  ns  ns | ns  ns  ns  ns  ↓  ns  ↓  ns  ↓  ns | | N/A  N/A  N/A  N/A  N/A  N/A  N/A  N/A  N/A  N/A | | N/A  N/A  N/A  N/A  N/A  N/A  N/A  N/A  N/A  N/A | Wireless EEG | min (light)  min (dark)  min (light)  min (dark)  min (light)  min (dark)  min (light)  min (dark)  min (light)  min (dark) | (Jyoti et al., 2015) |
|  | | | | | | | | | | | | |
|  | M/F | 5  12 | ↑↑↑  ↑↑ | ↓↓↓  ↓↓ | ns  ns | | N/A  N/A | | N/A  N/A | Wireless EEG | sec (24hr)  sec (24hr) | (Platt et al., 2011) |
|  | | | | | | | | | | | | |
| **OTHER MOUSE MODELS OF AD** | | | | | | | | | | | | |
|  | | | | | | | | | | | | |
| CVN-AD | F | 8-9 | N/A  N/A | N/A  N/A | N/A  N/A | | ns  ns  (p = 0.087 ↑) | | N/A  N/A | Locomotive monitoring | % (light)  % (dark) | (Nwafor et al., 2021) |
|  | | | | | | | | | | | | |
| P301S Tau | M | 3  6  9  11 | ns  ns  ns  ↑↑↑ | ns  ns  ns  ↓↓ | ns  ns  ↓↓  ↓↓↓↓ | | N/A  N/A  N/A  N/A | | N/A  N/A  N/A  N/A | EEG | min/hr (23hr)  min/hr (23hr)  min/hr (23hr)  min/hr (23hr) | (Holth et al., 2017) |
|  | | | | | | | | | | | | |
| rTg4510 | M | 20 (weeks)  24 (weeks)  28 (weeks)  32 (weeks)  36 (weeks)  40 (weeks)  44 (weeks) | ns  ns  ns  ↑  ns  ↑  ns  ↑  ns  ↑  ns  ↑  ns  ↑ | ns  ns  ns  ns  ns  ↓  ns  ↓  ns  ↓  ns  ns  ns  ↓ | ns  ns  ns  ns  ns  ns  ns  ns  ns  ns  ns  ns  ns  ns | | | N/A  N/A  N/A  N/A  N/A  N/A  N/A  N/A  N/A  N/A  N/A  N/A  N/A  N/A | N/A  N/A  N/A  N/A  N/A  N/A  N/A  N/A  N/A  N/A  N/A  N/A  N/A  N/A | EEG  EEG  EEG  EEG  EEG  EEG  EEG  EEG  EEG  EEG  EEG  EEG  EEG  EEG | min (light)  min (dark)  min (light)  min (dark)  min (light)  min (dark)  min (light)  min (dark)  min (light)  min (dark)  min (light)  min (dark)  min (light)  min (dark) | (Holton et al., 2020) |
|  | | | | | | | | | | | | |
| SAMP8 | M | 4 | ↑  ns | ↓  ns | ↓  ns | | N/A  N/A | | N/A  N/A | EEG  EEG | % (light)  % (dark) | (Beuckmann et al., 2021) |

* Tg comparisons with 9-mo WT mice (Roh et al., 2012)

F Female M Male

↑ Increase with p < 0.05 ↓ Decrease with p < 0.05

↑↑ Increase with p < 0.01 ↓↓ Decrease with p < 0.01

↑↑↑ Increase with p < 0.001 ↓↓↓ Decrease with p < 0.001

ns Not significant ↓↓↓↓ Decrease with p < 0.0001

N/A Not applicable

**References**

Beuckmann, C. T., Suzuki, H., Musiek, E. S., Ueno, T., Sato, T., Bando, M., et al. (2021). Evaluation of SAMP8 Mice as a Model for Sleep-Wake and Rhythm Disturbances Associated with Alzheimer’s Disease: Impact of Treatment with the Dual Orexin (Hypocretin) Receptor Antagonist Lemborexant. *J. Alzheimers Dis.*, 1-16.

Colby-Milley, J., Cavanagh, C., Jego, S., Breitner, J. C., Quirion, R., and Adamantidis, A. (2015). Sleep-wake cycle dysfunction in the TgCRND8 mouse model of Alzheimer’s disease: from early to advanced pathological stages. *PLoS One* 10, e0130177.

Duncan, M. J., Farlow, H., Tirumalaraju, C., Yun, D.-H., Wang, C., Howard, J. A., et al. (2019). Effects of the dual orexin receptor antagonist DORA-22 on sleep in 5XFAD mice. *Alzheimer's & Dementia: Translational Research & Clinical Interventions* 5, 70-80.

Filon, M. J., Wallace, E., Wright, S., Douglas, D. J., Steinberg, L. I., Verkuilen, C. L., et al. (2020). Sleep and diurnal rest-activity rhythm disturbances in a mouse model of Alzheimer’s disease. *Sleep* 43, zsaa087.

Holth, J. K., Mahan, T. E., Robinson, G. O., Rocha, A., and Holtzman, D. M. (2017). Altered sleep and EEG power in the P301S Tau transgenic mouse model. *Annals of clinical and translational neurology* 4, 180-190.

Holton, C., Hanley, N., Shanks, E., Oxley, P., McCarthy, A., Eastwood, B. J., et al. (2020). Longitudinal changes in EEG power, sleep cycles and behaviour in a tau model of neurodegeneration. *Alzheimers Res. Ther.* 12, 1-15.

Huitrón-Reséndiz, S., Sánchez-Alavez, M., Gallegos, R., Berg, G., Crawford, E., Giacchino, J. L., et al. (2002). Age-independent and age-related deficits in visuospatial learning, sleep–wake states, thermoregulation and motor activity in PDAPP mice. *Brain Res.* 928, 126-137.

Jyoti, A., Plano, A., Riedel, G., and Platt, B. (2010). EEG, Activity, and Sleep Architecture in a Transgenic AβPP swe/PSEN1 A246E Alzheimer's Disease Mouse. *J. Alzheimers Dis.* 22, 873-887.

---. (2015). Progressive age-related changes in sleep and EEG profiles in the PLB1Triple mouse model of Alzheimer’s disease. *Neurobiol. Aging* 36, 2768-2784.

Kent, B. A., Michalik, M., Marchant, E. G., Yau, K. W., Feldman, H. H., Mistlberger, R. E., et al. (2019). Delayed daily activity and reduced NREM slow-wave power in the APPswe/PS1dE9 mouse model of Alzheimer's disease. *Neurobiol. Aging* 78, 74-86.

Kent, B. A., Strittmatter, S. M., and Nygaard, H. B. (2018). Sleep and EEG power spectral analysis in three transgenic mouse models of Alzheimer’s disease: APP/PS1, 3xTgAD, and Tg2576. *J. Alzheimers Dis.* 64, 1325-1336.

Maezono, S. E. B., Kanuka, M., Tatsuzawa, C., Morita, M., Kawano, T., Kashiwagi, M., et al. (2020). Progressive changes in sleep and its relations to amyloid-β distribution and learning in single App knock-in mice. *Eneuro* 7.

Nwafor, D. C., Chakraborty, S., Jun, S., Brichacek, A. L., Dransfeld, M., Gemoets, D. E., et al. (2021). Disruption of metabolic, sleep, and sensorimotor functional outcomes in a female transgenic mouse model of Alzheimer’s disease. *Behav. Brain Res.* 398, 112983.

Oblak, A. L., Lin, P. B., Kotredes, K. P., Pandey, R. S., Garceau, D., Williams, H. M., et al. (2021). Comprehensive evaluation of the 5XFAD mouse model for preclinical testing applications: a MODEL-AD study. *Front. Aging Neurosci.* 13.

Platt, B., Drever, B., Koss, D., Stoppelkamp, S., Jyoti, A., Plano, A., et al. (2011). Abnormal cognition, sleep, EEG and brain metabolism in a novel knock-in Alzheimer mouse, PLB1. *PLoS One* 6, e27068.

Roh, J. H., Huang, Y., Bero, A. W., Kasten, T., Stewart, F. R., Bateman, R. J., et al. (2012). Disruption of the sleep-wake cycle and diurnal fluctuation of β-amyloid in mice with Alzheimer’s disease pathology. *Sci. Transl. Med.* 4, 150ra122-150ra122.

Schneider, F., Baldauf, K., Wetzel, W., and Reymann, K. (2014). Behavioral and EEG changes in male 5xFAD mice. *Physiol. Behav.* 135, 25-33.

Sethi, M., Joshi, S. S., Webb, R. L., Beckett, T. L., Donohue, K. D., Murphy, M. P., et al. (2015). Increased fragmentation of sleep–wake cycles in the 5XFAD mouse model of Alzheimer’s disease. *Neuroscience* 290, 80-89.

Van Erum, J., Van Dam, D., Sheorajpanday, R., and De Deyn, P. P. (2019). Sleep architecture changes in the APP23 mouse model manifest at onset of cognitive deficits. *Behav. Brain Res.* 373, 112089.

Wisor, J., Edgar, D., Yesavage, J., Ryan, H., McCormick, C., Lapustea, N., et al. (2005). Sleep and circadian abnormalities in a transgenic mouse model of Alzheimer’s disease: a role for cholinergic transmission. *Neuroscience* 131, 375-385.

Zhang, B., Veasey, S. C., Wood, M. A., Leng, L. Z., Kaminski, C., Leight, S., et al. (2005). Impaired rapid eye movement sleep in the Tg2576 APP murine model of Alzheimer's disease with injury to pedunculopontine cholinergic neurons. *The American journal of pathology* 167, 1361-1369.

Zhang, F., Zhong, R., Li, S., Fu, Z., Wang, R., Wang, T., et al. (2019). Alteration in sleep architecture and electroencephalogram as an early sign of Alzheimer's disease preceding the disease pathology and cognitive decline. *Alzheimer's & Dementia* 15, 590-597.
