# Supplementary material for: Progressive sleep disturbance in various transgenic mouse models of Alzheimer’s disease
Source: Front Aging Neurosci. 2023 May 19;15:1119810. doi: 10.3389/fnagi.2023.1119810 (PMC10235623; doi:10.3389/fnagi.2023.1119810)
Supplement: Supplementary file 3 [file Table_3.DOCX]

Supplementary table 3: Changes in vigilance state bout lengths in various mouse models of Alzheimer’s disease compared to age-matched controls

| **Mouse line** | **Sex** | **Age (months)** | **Wake length** | **NREM length** | **REM length** | **Sleep length** | **Epoch length** | **Bout length units (period)** | **References** |
| --- | --- | --- | --- | --- | --- | --- | --- | --- | --- |
|  | | | | | | | | | |
| **APP-BASED MOUSE MODELS OF AD** | | | | | | | | | |
|  | | | | | | | | | |
| APP23 | M | 3  6  12 | ns  ns  ns  ns  ns  ns  ns  ns  ns | ns  ns  ns  ns  ns  ns  ns  ns  ns | ns  ns  ns  ns  ns  ns  ns  ns  ns | N/A  N/A  N/A  N/A  N/A  N/A  N/A  N/A  N/A | 4 sec | Not specified (24hr)  Not specified (light)  Not specified (dark)  Not specified (24hr)  Not specified (light)  Not specified (dark)  Not specified (24hr)  Not specified (light)  Not specified (dark) | (Van Erum et al., 2019) |
|  | | | | | | | | | |
| App^NL-G-F/NL-G-F^ | M | 6  12 | ns  ↑ | ns  ns | ns  ↓↓ | N/A  N/A | 4 sec | sec (24hr)  sec (24hr) | (Maezono et al., 2020) |
|  | | | | | | | | | |
| PDAPP | F | 3-5  20-26 | N/A  N/A  N/A  N/A | ns  ns  ↑  ns | ns  ns  ↑  ns | N/A  N/A  N/A  N/A | N/A | min/episode (light)  min/episode (dark)  min/episode (light)  min/episode (dark) | (Huitrón-Reséndiz et al., 2002) |
|  | | | | | | | | | |
| Tg2576 | Not specified | 2  6  12 | ns  ns  ↓ | ns  ns  ns | ns  ↓  ↓ | N/A  N/A  N/A | 10 sec | min (24hr)  min (24hr)  min (24hr) | (Zhang et al., 2005) |
|  | | | | | | | | | |
| TgCRND8 | M | 3  7  11 | ↑  ↑  ns  ns  ↑  ns | ns  ns  ns  ns  ns  ns | ns  ns  ns  ns  ns  ns | N/A  N/A  N/A  N/A  N/A  N/A | 5 sec | sec (light)  sec (dark)  sec (light)  sec (dark)  sec (light)  sec (dark) | (Colby-Milley et al., 2015) |
|  | | | | | | | | | |
| **APP AND PSEN DOUBLE TRANSGENIC MOUSE MODELS OF AD** | | | | | | | | | |
|  | | | | | | | | | |
| 5XFAD | M/F | 4-4.5 | N/A  N/A  N/A | N/A  N/A  N/A | N/A  N/A  N/A | ↓  ↓  ↓ | N/A | sec (24hr)  sec (light)  sec (dark) | (Duncan et al., 2019) |
|  | | | | | | | | | |
|  | M      F | 4-6.5  4-6.5 | N/A  N/A  N/A  N/A  N/A  N/A | N/A  N/A  N/A  N/A  N/A  N/A | N/A  N/A  N/A  N/A  N/A  N/A | ↓↓  ↓↓  ↓↓  ↓↓↓  ↓↓  ↓↓↓ | "Signal features sensitive to the differences between the sleep and wake states are extracted from the short-time pressure signal segments, and classification is automatically performed every 2 s." | sec (24hr)  sec (light)  sec (dark)  sec (24hr)  sec (light)  sec (dark) | (Sethi et al., 2015) |
|  | | | | | | | | | |
|  | M  F | 10-11  10-11 | ns  ns | ns  ns | ns  ns | N/A  N/A | 5 sec (see Figure 3A in Oblak et al., 2021) | sec (period not specified)  sec (period not specified) | (Oblak et al., 2021) |
|  | | | | | | | | | |
| **OTHER MOUSE MODELS OF AD** | | | | | | | | | |
|  | | | | | | | | | |
| CVN-AD | F | 8-9 | N/A  N/A | N/A  N/A | N/A  N/A | ns  ns | 10 sec | Epochs (light)  Epochs (dark) | (Nwafor et al., 2021) |
|  | | | | | | | | | |
| P301S Tau | M | 3  6  9  11 | ns  ns  ↑↑  ↑↑ | ns  ns  ↑  ns | N/A  N/A  ns  ↓ | N/A  N/A  N/A  N/A | 10 sec | sec (23hr)  sec (23hr)  sec (23hr)  sec (23hr) | (Holth et al., 2017) |
|  | | | | | | | | | |
| rTg4510 | M | 20 (weeks)  24 (weeks)  28 (weeks)  32 (weeks)  36 (weeks)  40 (weeks)  44 (weeks) | N/A  N/A  N/A  N/A  N/A  N/A  N/A  N/A  N/A  N/A  N/A  N/A  N/A  N/A | N/A  N/A  N/A  N/A  N/A  N/A  N/A  N/A  N/A  N/A  N/A  N/A  N/A  N/A | ns  ns  ns  ↓  ns  ↓  ↓  ↓  ↓  ↓  ↓  ns  ↓  ↓ | ns  ns  ns  ns  ns  ns  ns  ns  ns  ns  ns  ns  ns  ns | 10 sec | min (light)  min (dark)  min (light)  min (dark)  min (light)  min (dark)  min (light)  min (dark)  min (light)  min (dark)  min (light)  min (dark)  min (light)  min (dark) | (Holton et al., 2020) |

F Female M Male

↑ Increase with p < 0.05 ↓ Decrease with p < 0.05

↑↑ Increase with p < 0.01 ↓↓ Decrease with p < 0.01

ns Not significant ↓↓↓ Decrease with p < 0.001

N/A Not applicable

**References**

Colby-Milley, J., Cavanagh, C., Jego, S., Breitner, J. C., Quirion, R., and Adamantidis, A. (2015). Sleep-wake cycle dysfunction in the TgCRND8 mouse model of Alzheimer’s disease: from early to advanced pathological stages. *PLoS One* 10, e0130177.

Duncan, M. J., Farlow, H., Tirumalaraju, C., Yun, D.-H., Wang, C., Howard, J. A., et al. (2019). Effects of the dual orexin receptor antagonist DORA-22 on sleep in 5XFAD mice. *Alzheimer's & Dementia: Translational Research & Clinical Interventions* 5, 70-80.

Holth, J. K., Mahan, T. E., Robinson, G. O., Rocha, A., and Holtzman, D. M. (2017). Altered sleep and EEG power in the P301S Tau transgenic mouse model. *Annals of clinical and translational neurology* 4, 180-190.

Holton, C., Hanley, N., Shanks, E., Oxley, P., McCarthy, A., Eastwood, B. J., et al. (2020). Longitudinal changes in EEG power, sleep cycles and behaviour in a tau model of neurodegeneration. *Alzheimers Res. Ther.* 12, 1-15.

Huitrón-Reséndiz, S., Sánchez-Alavez, M., Gallegos, R., Berg, G., Crawford, E., Giacchino, J. L., et al. (2002). Age-independent and age-related deficits in visuospatial learning, sleep–wake states, thermoregulation and motor activity in PDAPP mice. *Brain Res.* 928, 126-137.

Maezono, S. E. B., Kanuka, M., Tatsuzawa, C., Morita, M., Kawano, T., Kashiwagi, M., et al. (2020). Progressive changes in sleep and its relations to amyloid-β distribution and learning in single App knock-in mice. *Eneuro* 7.

Nwafor, D. C., Chakraborty, S., Jun, S., Brichacek, A. L., Dransfeld, M., Gemoets, D. E., et al. (2021). Disruption of metabolic, sleep, and sensorimotor functional outcomes in a female transgenic mouse model of Alzheimer’s disease. *Behav. Brain Res.* 398, 112983.

Oblak, A. L., Lin, P. B., Kotredes, K. P., Pandey, R. S., Garceau, D., Williams, H. M., et al. (2021). Comprehensive evaluation of the 5XFAD mouse model for preclinical testing applications: a MODEL-AD study. *Front. Aging Neurosci.* 13.

Sethi, M., Joshi, S. S., Webb, R. L., Beckett, T. L., Donohue, K. D., Murphy, M. P., et al. (2015). Increased fragmentation of sleep–wake cycles in the 5XFAD mouse model of Alzheimer’s disease. *Neuroscience* 290, 80-89.

Van Erum, J., Van Dam, D., Sheorajpanday, R., and De Deyn, P. P. (2019). Sleep architecture changes in the APP23 mouse model manifest at onset of cognitive deficits. *Behav. Brain Res.* 373, 112089.

Zhang, B., Veasey, S. C., Wood, M. A., Leng, L. Z., Kaminski, C., Leight, S., et al. (2005). Impaired rapid eye movement sleep in the Tg2576 APP murine model of Alzheimer's disease with injury to pedunculopontine cholinergic neurons. *The American journal of pathology* 167, 1361-1369.
