# Supplementary material for: Progressive sleep disturbance in various transgenic mouse models of Alzheimer’s disease
Source: Front Aging Neurosci. 2023 May 19;15:1119810. doi: 10.3389/fnagi.2023.1119810 (PMC10235623; doi:10.3389/fnagi.2023.1119810)
Supplement: Supplementary file 4 [file Table_4.DOCX]

Supplementary table 4: Changes in vigilance state bout counts in various mouse models of Alzheimer’s disease compared to age-matched controls

| **Mouse line** | **Sex** | **Age (months)** | **Wake count** | **NREM count** | **REM count** | **Sleep count** | **Epoch length** | **Bout count units (period)** | **References** |
| --- | --- | --- | --- | --- | --- | --- | --- | --- | --- |
|  | | | | | | | | | |
| **APP-BASED MOUSE MODELS OF AD** | | | | | | | | | |
|  | | | | | | | | | |
| App^NL-G-F/NL-G-F^ | M | 6  12 | ↓  ns | ↓  ↓ | ns  ns | N/A  N/A | 4 sec | # of episodes (24hr)  # of episodes (24hr) | (Maezono et al., 2020) |
|  | | | | | | | | | |
| PDAPP | F | 3-5  20-26 | N/A  N/A  N/A  N/A | ns  ns  ns  ns | ns  ns  ns  ↓ | N/A  N/A  N/A  N/A | Un-known | # of episodes (light)  # of episodes (dark)  # of episodes (light)  # of episodes (dark) | (Huitrón-Reséndiz et al., 2002) |
|  | | | | | | | | | |
| **OTHER MOUSE MODELS OF AD** | | | | | | | | | |
|  | | | | | | | | | |
| CVN-AD | F | 8-9 | N/A  N/A | N/A  N/A | N/A  N/A | ns  ↑ | 10 sec | # of episodes (light)  # of episodes (dark) | (Nwafor et al., 2021) |
|  | | | | | | | | | |
| P301S Tau | M | 3  6  9  11 | N/A  N/A | ns  ns  ↓↓  ↓ | ns  ns  ↓  ↓↓↓↓ | N/A  N/A  N/A  N/A | 10 sec | # of episodes (23hr)  # of episodes (23hr)  # of episodes (23hr)  # of episodes (23hr) | (Holth et al., 2017) |
|  |  |  | ↓↓  ↓ |  |  |  |  |  |  |
|  | | | | | | | | | |
| rTg4510 | M | 20 (weeks)  24 (weeks)  28 (weeks)  32 (weeks)  36 (weeks)  40 (weeks)  44 (weeks) | N/A  N/A  N/A  N/A  N/A  N/A  N/A  N/A  N/A  N/A  N/A  N/A  N/A  N/A | N/A  N/A  N/A  N/A  N/A  N/A  N/A  N/A  N/A  N/A  N/A  N/A  N/A  N/A | ns  ns  ns  ↓  ns  ↓  ns  ↓  ns  ↓  ↓  ns  ↓  ↓ | ns  ns  ns  ns  ns  ns  ns  ns  ns  ns  ns  ns  ns  ns | 10 sec | # of episodes (light)  # of episodes (dark)  # of episodes (light)  # of episodes (dark)  # of episodes (light)  # of episodes (dark)  # of episodes (light)  # of episodes (dark)  # of episodes (light)  # of episodes (dark)  # of episodes (light)  # of episodes (dark)  # of episodes (light)  # of episodes (dark) | (Holton et al., 2020) |

F Female M Male

↑ Increase with p < 0.05 ↓ Decrease with p < 0.05

ns, Not significant ↓↓ Decrease with p < 0.01

N/A, Not applicable ↓↓↓↓ Decrease with p < 0.0001

**References (to be matched with manuscript text)**

Holth, J. K., Mahan, T. E., Robinson, G. O., Rocha, A., and Holtzman, D. M. (2017). Altered sleep and EEG power in the P301S Tau transgenic mouse model. *Annals of clinical and translational neurology* 4, 180-190.

Holton, C., Hanley, N., Shanks, E., Oxley, P., McCarthy, A., Eastwood, B. J., et al. (2020). Longitudinal changes in EEG power, sleep cycles and behaviour in a tau model of neurodegeneration. *Alzheimers Res. Ther.* 12, 1-15.

Huitrón-Reséndiz, S., Sánchez-Alavez, M., Gallegos, R., Berg, G., Crawford, E., Giacchino, J. L., et al. (2002). Age-independent and age-related deficits in visuospatial learning, sleep–wake states, thermoregulation and motor activity in PDAPP mice. *Brain Res.* 928, 126-137.

Maezono, S. E. B., Kanuka, M., Tatsuzawa, C., Morita, M., Kawano, T., Kashiwagi, M., et al. (2020). Progressive changes in sleep and its relations to amyloid-β distribution and learning in single App knock-in mice. *Eneuro* 7.

Nwafor, D. C., Chakraborty, S., Jun, S., Brichacek, A. L., Dransfeld, M., Gemoets, D. E., et al. (2021). Disruption of metabolic, sleep, and sensorimotor functional outcomes in a female transgenic mouse model of Alzheimer’s disease. *Behav. Brain Res.* 398, 112983.
