# Supplementary material for: Progressive sleep disturbance in various transgenic mouse models of Alzheimer’s disease
Source: Front Aging Neurosci. 2023 May 19;15:1119810. doi: 10.3389/fnagi.2023.1119810 (PMC10235623; doi:10.3389/fnagi.2023.1119810)
Supplement: Supplementary file 5 [file Table_5.DOCX]

Supplementary table 5: Changes in power spectra in various mouse models of Alzheimer’s disease compared to age-matched controls

| **Mouse line** | **Sex** | **Age (months)** | **Wake Delta** | **NREM Delta** | **REM Delta** | **Delta Range** | **NREM Theta** | **REM Theta** | **Theta Range** | **Units** | **Region** | **Rec. Duration** | **Ref.** |
| --- | --- | --- | --- | --- | --- | --- | --- | --- | --- | --- | --- | --- | --- |
|  | | | | | | | | | | | | | |
| **APP-BASED MOUSE MODELS OF AD** | | | | | | | | | | | | | |
|  | | | | | | | | | | | | | |
| APP23 | M | 3  6  12 | ns  ↓  ↓ | ns  ↓  ↓ | ns  ns  ns | 0.5-3.5 Hz | ns  ns  ns | ns  ns  ns | 3.5-7 Hz | Relative power (%) | Parietal | 24 hrs  24 hrs  24 hrs | (Van Erum et al., 2019) |
|  | | | | | | | | | | | | | |
| App^NL-G-F/NL-G-F^ | M | 6  12 | ↑↑  ↑↑↑ | ns  ↑↑↑ | ↑  ↑↑ | 1-4 Hz | ↓  ↓↓↓ | ns  ↓ | 7-10 Hz | Normalized power | Parietal cortex and cerebellum | 24 hrs  24 hrs | (Maezono et al., 2020) |
|  | | | | | | | | | | | | | |
| J20 | Not specified | 11-12 | N/A | "↓" | N/A | 0.5-4 Hz | "↑" | N/A | 5-9 Hz | EEG power (A.U.) | Frontal and parietal bones | 3 days  3 days | (Filon et al., 2020) |
|  | | | | | | | | | | | | | |
| Tg2576 | M  F | 8  11  15  17  22 | N/A  N/A  N/A  N/A | "unclear"  "unclear"  "unclear"  "↑" | N/A  N/A  N/A  N/A  N/A  N/A  N/A  N/A | 1-4 Hz | "unclear"  "unclear"  "unclear"  "↑" | N/A  N/A  N/A  N/A  N/A  N/A  N/A  N/A | 6-9 Hz | NREM EEG power μV^2/Hz | Not specified | 24 hrs  24 hrs  24 hrs  24 hrs | (Wisor et al., 2005) |
|  |  |  | N/A  N/A  N/A  N/A | ↓  ns  ↑  ↑ |  |  | N/A  N/A  N/A  N/A |  |  | Normalized delta power (ZT0-3) | Not specified | ZT0-3  ZT3-6  ZT6-9  ZT9-12 |  |
|  | | | | | | | | | | | | | |
|  | M/F | 12 | ns  ↓ | ns  ns | ns  ns | 0.1-4 Hz | ns  ns | ns  ns | 4-8 Hz | Power (%)  Power (%) | Frontal  Parietal | 24 hrs  24 hrs | (Kent et al., 2018) |
|  | | | | | | | | | | | | | |
| TgCRND8 | M | 3  7  11 | ↓↓  ns  ns | ns  ns  ns | N/A  N/A  N/A | 0.5-4.5 Hz | N/A  N/A  N/A | ns  ↓  ↓ | 6-10 Hz | Normalized power (V^2) | Frontal and parietal | 24 hrs  24 hrs  24 hrs | (Colby-Milley et al., 2015) |
|  | | | | | | | | | | | | | |
| **APP AND PSEN DOUBLE TRANSGENIC MOUSE MODELS OF AD** | | | | | | | | | | | | | |
|  | | | | | | | | | | | | | |
| 5XFAD | M | 3  6 | N/A  N/A | N/A  N/A | N/A  N/A | 0.5-4 Hz | N/A  N/A | N/A  N/A | 4-8 Hz | Intensity (μVpp in %) | Parietal cortex | 1 hr x 3 days  1 hr x 3 days | (Schreiner and Rasch, 2015) |
|  | | | | | | | | | | | | | |
|  | M  F | 10-11  10-11 | ns  ns | ns  ns | ns  ns | 0.5-4 Hz | ns  ns | ns  ns | 4-8 Hz | Power (%)  Power (%) | Cortical  Cortical | 72 hrs  72 hrs | (Oblak et al., 2021) |
|  | | | | | | | | | | | | | |
| AβPP^swe^/PS1^∆E9^ | M/F | 8-10 | N/A  N/A | ns  ↓ | N/A  N/A | 0.1-4 Hz | ns  ns | N/A  N/A | 4-8 Hz | Power (%)  Power (%) | Frontal  Parietal | 24 hrs  24 hrs | (Kent et al., 2018) |
|  | | | | | | | | | | | | | |
|  | F | 12 | ns | ↓↓ | N/A | 0.1-4 Hz | ns | N/A | 4-8 Hz | Power (%) | Not specified | 72 hrs | (Kent et al., 2019) |
|  | | | | | | | | | | | | | |
| **APP. PSEN AND TAU TRANSGENIC MOUSE MODELS OF AD** | | | | | | | | | | | | | |
|  | | | | | | | | | | | | | |
| 3xTgAD | M/F | 18 | N/A | N/A | N/A | 0.1-4 Hz | N/A | N/A | 4-8 Hz | N/A | N/A | 24 hrs | (Kent et al., 2018) |
|  | | | | | | | | | | | | | |
| PLB1 | M/F  M/F | 5  9  13  17  21  5  9  13  17  21 | ns  ns  ns  ns  ns  ns  ns  ns  ↑↑  ↑↑ | ns  ns  ns  ns  ns  ↑↑  ns  ↑↑  ↑↑  ns | ns  ↓↓  ns  ns  ns  ns  ns  ns  ns  ns | 0.5-5 Hz | ns  ns  ns  ↑  ↑↑  ns  ns  ns  ↑  ↑↑↑ | ↑  ns  ns  ns  ns  ns  ns  ns  ns  ↑↑↑ | 5-9 Hz | Normalized power      Normalized power | Prefrontal cortex      Parietal cortex/hippo. | 5 days  5 days  5 days  5 days  5 days  5 days  5 days  5 days  5 days  5 days | (Jyoti et al., 2015) |
|  | | | | | | | | | | | | | |
|  | M/F  M/F | 5  12  5  12 | ↑↑  ns  ns  ns | ↑  ns  ns  ↑↑ | ns  ns  ↑↑  ns | 0.5-5 Hz | ns  ↑↑  ns  ↑↑↑ | ↑  ns  ns  ns | 5-9 Hz | Normalized power  Normalized power | Prefrontal cortex  Parietal cortex | 24 hrs  24 hrs  24 hrs  24 hrs | (Platt et al., 2011) |
|  | | | | | | | | | | | | | |
| **OTHER MOUSE MODELS OF AD** | | | | | | | | | | | | | |
|  | | | | | | | | | | | | | |
| P301S Tau | M | 3  6  9  11 | ns  ns  ns  "↓" | ns  "↑"  "↑"  "↓" | ns  ns  ns  ns | 1-4 Hz | ns  ns  "↑"  ns | ns  ns  "↑"  "↓" | 4-8 Hz | Power (μV^2) | Right frontal and parietal bone | 23 hrs  23 hrs  23 hrs  23 hrs | (Holth et al., 2017) |
|  | | | | | | | | | | | | | |
| rTg4510 | M | 20 (weeks)  24 (weeks)  28 (weeks)  32 (weeks)  36 (weeks)  40 (weeks)  44 (weeks) | N/A  N/A  N/A  N/A  N/A  N/A  N/A  N/A  N/A  N/A  N/A  N/A  N/A  N/A | ns  ns  ↓  ↓  ↓  ↓  ↓  ↓  ↓  ↓  ↓  ↓  ↓  ↓ | N/A  N/A  N/A  N/A  N/A  N/A  N/A  N/A  N/A  N/A  N/A  N/A  N/A  N/A | 0.1-4 Hz | N/A  N/A  N/A  N/A  N/A  N/A  N/A  N/A  N/A  N/A  N/A  N/A  N/A  N/A | N/A  N/A  N/A  N/A  N/A  N/A  N/A  N/A  N/A  N/A  N/A  N/A  N/A  N/A | 5.1-9 Hz | Power mean μV^2/10s | Frontal and occipital | 1 week (light)  1 week (dark)  1 week (light)  1 week (dark)  1 week (light)  1 week (dark)  1 week (light)  1 week (dark)  1 week (light)  1 week (dark)  1 week (light)  1 week (dark)  1 week (light)  1 week (dark) | (Holton et al., 2020) |

" " Indicators of significance are approximately or unclear, or ranges are unspecified in article (Wisor et al., 2005; Holth et al., 2017)

F Female M Male

↑ Increase with p < 0.05 ↓ Decrease with p < 0.05

↑↑ Increase with p < 0.01 ↓↓ Decrease with p < 0.01

ns, Not significant ↓↓↓ Decrease with p < 0.001

N/A, Not applicable

**References**

Colby-Milley, J., Cavanagh, C., Jego, S., Breitner, J. C., Quirion, R., and Adamantidis, A. (2015). Sleep-wake cycle dysfunction in the TgCRND8 mouse model of Alzheimer’s disease: from early to advanced pathological stages. *PLoS One* 10, e0130177.

Filon, M. J., Wallace, E., Wright, S., Douglas, D. J., Steinberg, L. I., Verkuilen, C. L., et al. (2020). Sleep and diurnal rest-activity rhythm disturbances in a mouse model of Alzheimer’s disease. *Sleep* 43, zsaa087.

Holth, J. K., Mahan, T. E., Robinson, G. O., Rocha, A., and Holtzman, D. M. (2017). Altered sleep and EEG power in the P301S Tau transgenic mouse model. *Annals of clinical and translational neurology* 4, 180-190.

Holton, C., Hanley, N., Shanks, E., Oxley, P., McCarthy, A., Eastwood, B. J., et al. (2020). Longitudinal changes in EEG power, sleep cycles and behaviour in a tau model of neurodegeneration. *Alzheimers Res. Ther.* 12, 1-15.

Jyoti, A., Plano, A., Riedel, G., and Platt, B. (2015). Progressive age-related changes in sleep and EEG profiles in the PLB1Triple mouse model of Alzheimer’s disease. *Neurobiol. Aging* 36, 2768-2784.

Kent, B. A., Michalik, M., Marchant, E. G., Yau, K. W., Feldman, H. H., Mistlberger, R. E., et al. (2019). Delayed daily activity and reduced NREM slow-wave power in the APPswe/PS1dE9 mouse model of Alzheimer's disease. *Neurobiol. Aging* 78, 74-86.

Kent, B. A., Strittmatter, S. M., and Nygaard, H. B. (2018). Sleep and EEG power spectral analysis in three transgenic mouse models of Alzheimer’s disease: APP/PS1, 3xTgAD, and Tg2576. *J. Alzheimers Dis.* 64, 1325-1336.

Maezono, S. E. B., Kanuka, M., Tatsuzawa, C., Morita, M., Kawano, T., Kashiwagi, M., et al. (2020). Progressive changes in sleep and its relations to amyloid-β distribution and learning in single App knock-in mice. *Eneuro* 7.

Oblak, A. L., Lin, P. B., Kotredes, K. P., Pandey, R. S., Garceau, D., Williams, H. M., et al. (2021). Comprehensive evaluation of the 5XFAD mouse model for preclinical testing applications: a MODEL-AD study. *Front. Aging Neurosci.* 13.

Platt, B., Drever, B., Koss, D., Stoppelkamp, S., Jyoti, A., Plano, A., et al. (2011). Abnormal cognition, sleep, EEG and brain metabolism in a novel knock-in Alzheimer mouse, PLB1. *PLoS One* 6, e27068.

Schreiner, T., and Rasch, B. (2015). Boosting vocabulary learning by verbal cueing during sleep. *Cereb. Cortex* 25, 4169-4179.

Van Erum, J., Van Dam, D., Sheorajpanday, R., and De Deyn, P. P. (2019). Sleep architecture changes in the APP23 mouse model manifest at onset of cognitive deficits. *Behav. Brain Res.* 373, 112089.

Wisor, J., Edgar, D., Yesavage, J., Ryan, H., McCormick, C., Lapustea, N., et al. (2005). Sleep and circadian abnormalities in a transgenic mouse model of Alzheimer’s disease: a role for cholinergic transmission. *Neuroscience* 131, 375-385.
